# Membrane Sterol Composition in *Arabidopsis thaliana* Affects Root Elongation via Auxin Biosynthesis

**DOI:** 10.3390/ijms22010437

**Published:** 2021-01-04

**Authors:** Meng Wang, Panpan Li, Yao Ma, Xiang Nie, Markus Grebe, Shuzhen Men

**Affiliations:** 1 Department of Plant Biology and Ecology, College of Life Sciences, Nankai University and Tianjin Key Laboratory of Protein Sciences, Tianjin 300071, China; wangmm113@126.com (M.W.); panda411190351@126.com (P.L.); yaoma@mail.nankai.edu.cn (Y.M.); niexiang@nankai.edu.cn (X.N.); 2 Institute of Biochemistry and Biology, Plant Physiology, University of Potsdam, D-14476 Potsdam-Golm, Germany; markus.grebe@uni-potsdam.de

**Keywords:** *Arabidopsis thaliana*, auxin, auxin biosynthesis, cycloeucalenol, CPI1, sitosterol, sterol

## Abstract

Plant membrane sterol composition has been reported to affect growth and gravitropism via polar auxin transport and auxin signaling. However, as to whether sterols influence auxin biosynthesis has received little attention. Here, by using the sterol biosynthesis mutant *cyclopropylsterol isomerase1-1* (*cpi1-1*) and sterol application, we reveal that cycloeucalenol, a CPI1 substrate, and sitosterol, an end-product of sterol biosynthesis, antagonistically affect auxin biosynthesis. The short root phenotype of *cpi1-1* was associated with a markedly enhanced auxin response in the root tip. Both were neither suppressed by mutations in polar auxin transport (PAT) proteins nor by treatment with a PAT inhibitor and responded to an auxin signaling inhibitor. However, expression of several auxin biosynthesis genes *TRYPTOPHAN AMINOTRANSFERASE OF ARABIDOPSIS1* (*TAA1*) was upregulated in *cpi1-1.* Functionally, *TAA1* mutation reduced the auxin response in *cpi1-1* and partially rescued its short root phenotype. In support of this genetic evidence, application of cycloeucalenol upregulated expression of the auxin responsive reporter *DR5:GUS* (β-glucuronidase) and of several auxin biosynthesis genes, while sitosterol repressed their expression. Hence, our combined genetic, pharmacological, and sterol application studies reveal a hitherto unexplored sterol-dependent modulation of auxin biosynthesis during Arabidopsis root elongation.

## 1. Introduction

Sterols are essential membrane components in all eukaryotes [1]. Plants have a complex sterol composition in which sitosterol, campesterol, stigmasterol, and isofucosterol are predominant [2,3,4,5]. Besides, sterol biosynthetic intermediates (SBIs), such as cycloartenol, 24-methylene cycloartanol, cycloeucalenol, obtusifoliol, 24-ethylidene lophenol, are present as minor sterol compounds [2,6,7,8,9,10] (Supplemental Figure S1). Campesterol is the precursor for the biosynthesis of brassinosteroids (BRs), which is the only steroid hormone identified in plants so far. Whereas the dwarf phenotype of downstream sterol biosynthesis mutants, such as *dwarf7* (*dwf7)/sterol1* (*ste1*), *dwf5*, and *dwf1/diminuto (dim)* could be rescued by BRs [11,12,13,14], the defect of root or hypocotyl elongation in upstream mutants such as *fackel* (*fk*)/*hydra2* (*hyd2*), *hyd1*, *sterol methyltransferase1* (*smt1*), *cyp51A2*, *cyclopropylsterol isomerase1-1 (cpi1-1)*, *sterol 4α-methyl oxidase2-1* (*smo2-1*) *smo2-2/+*, and *smo1-1 smo1-2/+* could not be rescued by BRs [8,15,16,17,18,19,20,21,22], suggesting essential roles of sterols per se in cell division and cell expansion.

The upstream sterol mutants often display defects in auxin response and polar auxin transport (PAT). The *fk/hyd2* and *hyd1* mutants showed enhanced responses to auxin, and their short root and short leaf petiole phenotypes could be partially rescued by blocking auxin signaling [23]. The *cotyledon vascular patterning1* (*cvp1*) mutant showed weak auxin insensitivity, and could enhance the auxin resistance of *transport inhibitor resistant1* (*tir1*) and *auxin resistance1-3* (*axr1-3*) mutants [9,24]. The *smt1*, *cpi1-1*, *fk/hyd2, hyd1, smt2 smt3, smo2-1 smo2-2,* and *smo1-1 smo1-2* mutants displayed aberrant localizations of PIN-FORMED (PIN) auxin efflux carriers in seedling root or embryo [8,19,21,22,25,26,27]. It has been demonstrated that sterol endocytosis shares a common route with PIN2 recycling [28], and correct sterol composition was required for endocytosis of PIN2 for the establishment of its polar localization after cytokinesis [8]. Recycling of PIN2 from brefeldin A (BFA) toxin-induced endocytic agglomerates to the plasma membrane was inhibited in the *smt2 smt3* mutant [26]. Sterols were also required for auxin inhibition of PIN2 endocytosis [24]. Zhang et al. (2016) demonstrated that the embryo-lethal phenotype of the *smo2-1 smo2-2* double mutant could be partially rescued by endogenous or exogenous auxin supplementation, while the dwarf phenotype of the *smo2-1 smo2-2/+* mutant was completely rescued by endogenous auxin overproduction [21]. This finding implies that not only PAT was defective in sterol mutants, but auxin biosynthesis might be abnormal as well. Indeed, recently Song et al. (2019) showed that in the *smo1-1 smo1-2* embryos, expression of the *TRYPTOPHAN AMINOTRANSFERASE OF ARABIDOPSIS1* (*TAA1*) and *YUCCA9* (*YUC9*) auxin biosynthesis pathway genes was abnormal [22]. However, how sterols modulate auxin biosynthesis remains unclear.

In this study, we used the *cpi1-1* mutant to dissect the function of sterols in modulating auxin biosynthesis. The *CPI1* gene encodes the cyclopropylsterol isomerase, which is responsible for opening the 9β,19-cyclopropane ring of cycloeucalenol and converting it into obtusifoliol [29,30] (Supplemental Figure S1B). The *cpi1-1* mutant exhibits a short root, agravitropism, and stomatal developmental defects [8,31]. Expression of the auxin responsive reporter *DR5:GUS* [32,33] in the *cpi1-1* root tip was markedly enhanced and expanded to the lateral root cap and epidermal cells [8]. We found that expression of auxin biosynthesis genes including *TAA1*, *YUC8*, and *YUC9* was upregulated in *cpi1-1*. *TAA1* mutation reduced *DR5:GUS* expression in *cpi1-1* root tip and partially rescued the short root phenotype of *cpi1-1*. Since sterol composition in *cpi1-1* was markedly altered, with accumulation of cycloeucalenol at the expense of almost complete reduction of the major sterols such as sitosterol, 24-methylcholesterol (campesterol), and stigmasterol [8], we analyzed the effect of these sterols on the expression of *DR5:GUS* and auxin biosynthetic genes and on the root length of wild type (WT) and *cpi1-1*. We found that cycloeucalenol upregulated the expression of *DR5:GUS* and auxin biosynthetic genes, whereas sitosterol repressed the expression of these genes and partially rescued the short root phenotype of *cpi1-1*.

## 2. Results

### 2.1. An Increased Auxin Response in cpi1-1 is Further Enhanced by Defective Polar Auxin Transport

**Figure 1:**
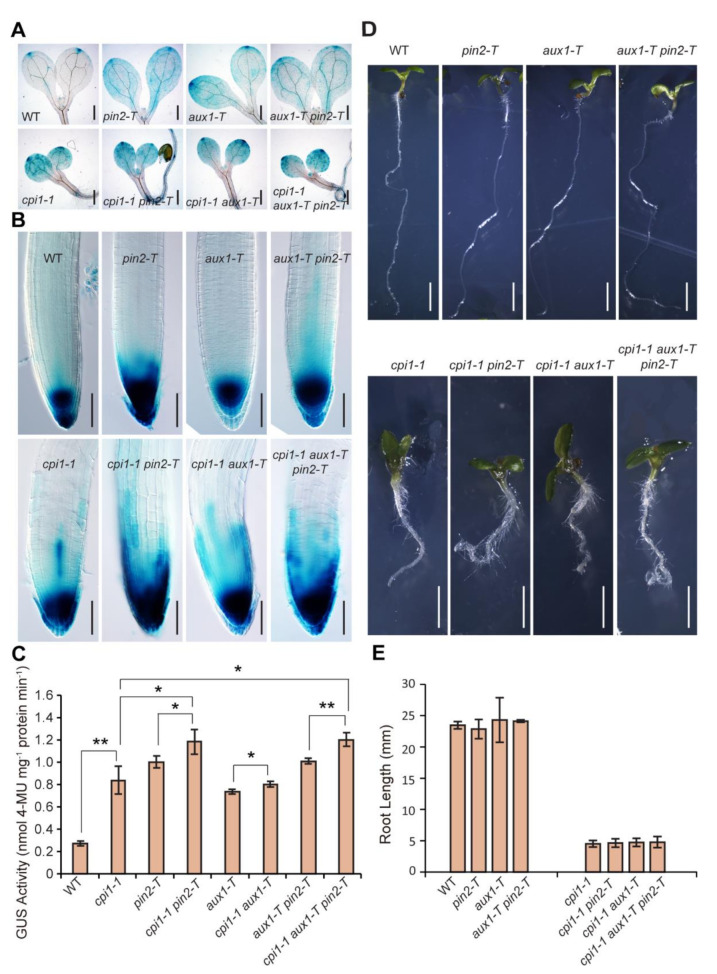
Auxin response reporter expression in and genetic interactions between *cpi1-1*, *pin2-T*, and *aux1-T* mutant combinations. (**A**,**B**) *DR5:GUS* expression patterns in 5-day-old WT, *cpi1-1*, *pin2-T*, *cpi1-1 pin2-T*, *aux1-T*, *cpi1-1 aux1-T*, *aux1-T pin2-T*, and *cpi1-1 aux1-T pin2-T* cotyledons (**A**) and seedling roots (**B**). Shown are representative images of three independent experiments (employing 10 to 16 seedlings per experiment); (**C**) Quantitative *DR5:GUS* activity assay in 5-day-old seedlings. The presented data are means ± SD of *n* = 3 independent experiments. * *p* < 0.05, ** *p* < 0.01 (one-way ANOVA with Tukey’s test); (**D**,**E**) Phenotypes (**D**) and root length (**E**) of 7-day-old seedlings. The presented data are means ± SD of *n* = 3 independent experiments (employing 9 to 37 seedlings per experiment). No significant difference between *cpi1-1* single and *cpi1-1 pin2-T* and *cpi1-1 aux1-T* double and *cpi1-1 aux1-T pin2-T* triple mutants by Student’s *t*-test (one-tailed, two-sample equal variance, *p* < 0.05). Bars = 400 μm in (**A**), 100 μm in (**B**), and 2 mm in (**D**).

We initially observed the auxin response reporter *DR5:GUS* whose expression correlates with auxin levels [32,33]. Compared to the WT, *cpi1-1* displayed markedly enhanced and ectopic *DR5:GUS* expression (Figure 1A,B) [8]. In WT cotyledons, *DR5:GUS* expression was detected at the tip, whereas in *cpi1-1*, *DR5:GUS* expression was expanded to the whole cotyledon where the cotyledon tips and the vasculature revealed strong GUS signals (Figure 1A). In the WT root tip, *DR5:GUS* expression was detected in the stem cell niche and columella root cap cells, whereas in the *cpi1-1* root tip, *DR5:GUS* expression was markedly enhanced and expanded to the lateral root cap and epidermal cells (Figure 1B). In addition, roots of 2-week-old *cpi1-1* seedlings displayed more lateral root primordia (which were marked by strong *DR5:GUS* signal) and lateral roots than that of the WT (Supplemental Figure S2). Polar localization of PIN2 has previously been reported to be defective in *cpi1-1* root epidermal and cortex cells [8]. To address whether elevated auxin activity in *cpi1-1* was caused by defective PAT, we crossed the loss-of-function *pin2-T* [8], *aux1-T* [34], and *aux1-T pin2-T* [35,36] alleles to *cpi1-1*, and compared the *DR5:GUS* expression patterns among the single, double, and triple mutants. The *cpi1-1 pin2-T* double mutant displayed higher *DR5:GUS* activity than either *cpi1-1* or *pin2-T*, whereas there was no obvious difference of *DR5:GUS* activity between *cpi1-1 aux1-T* and *cpi1-1* (Figure 1A–C). Similar to the *cpi1-1 pin2-T* double mutant, the *cpi1-1 aux1-T pin2-T* triple mutant displayed stronger *DR5:GUS* activity than either *cpi1-1* or *aux1-T pin2-T* (Figure 1A–C). However, there was no significant difference of the root length between *cpi1-1* single, *cpi1-1 pin2-T* and *cpi1-1 aux1-T* double, and *cpi1-1 aux1-T pin2-T* triple mutants (Figure 1D,E). These results indicate that *CPI1* and *PIN2* mutation have additive effects in *DR5:GUS* activity in the root tip, but there is not an additive effect on root elongation.

Previously Men et al. (2008) had shown that *PIN2* transcript level was not affected in *cpi1-1* by using semi-quantitative reverse transcription PCR (RT-PCR) [8]. We checked the expression of other *PIN* genes in *cpi1-1* by crossing *ProPIN1:GUS*, *ProPIN3:GUS*, *ProPIN4:GUS*, and *ProPIN7:GUS* to *cpi1-1*. Compared to WT, slightly enhanced *ProPIN1:GUS* and *ProPIN3:GUS* staining were observed in *cpi1-1* (Supplemental Figure S3), while expression of *ProPIN4:GUS* and *ProPIN7:GUS* was not obviously different (Supplemental Figure S4A–D). We also examined PIN7 expression by observing ProPIN7:PIN7-GFP (PIN7-GFP) fluorescence in seedling root tips. Consistently, no obvious differential expression of PIN7-GFP was detected between *cpi1-1* and WT (Supplemental Figure S4E–J). To gain further evidence, RT-quantitative PCR (RT-qPCR) was performed on 7-day-old WT and *cpi1-1* seedlings. Consistent with the GUS staining results, relative transcript levels of *PIN1* and *PIN3* were increased in *cpi1-1* mutants (Supplemental Figure S4K).

**Figure 2:**
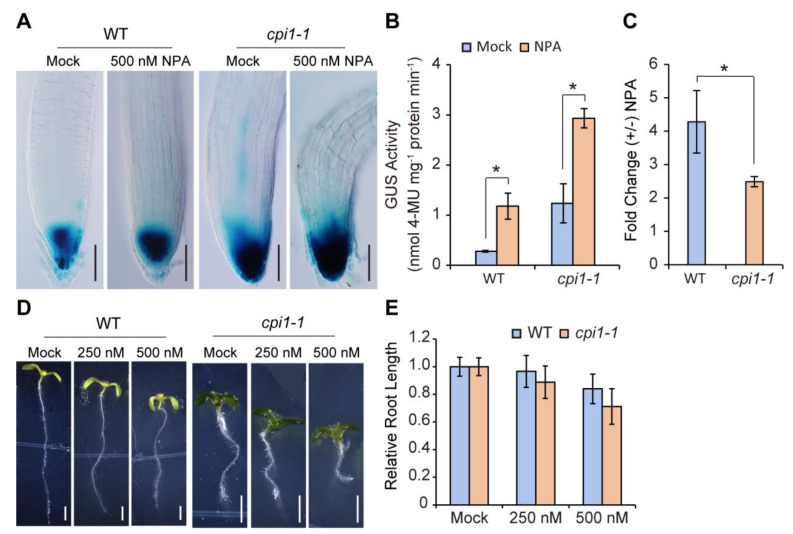
The auxin transport inhibitor 1-*N*-naphthylphthalamic acid (NPA) increases the *DR5:GUS* signal in WT and *cpi1-1* root tips. (**A**) NPA increases the *DR5:GUS* signal in 5-day-old WT and *cpi1-1* seedling root tips. Shown are representative images of *n* = 3 independent experiments, employing 7 to 21 seedlings per experiment; (**B**) Quantitative GUS activity assay in 5-day-old WT and *cpi1-1* seedlings expressing *DR5:GUS*; (**C**) Fold change of *DR5:GUS* activity in WT and *cpi1-1* seedlings upon NPA treatment. The data presented in (**B**,**C**) are means ± SD of *n* = 3 independent experiments. * *p* < 0.05 (Student’s *t*-test, one-tailed, two-sample equal variance); (**D**,**E**) Phenotypes (**D**) and relative root length (**E**) of WT and *cpi1-1* seedlings after NPA treatment for 7 days. The data presented in (**E**) are means ± SD of *n* = 3 independent experiments (employing 10 to 45 seedlings per experiment). No significant difference between WT and *cpi1-1* mutant by Student’s *t*-test (one-tailed, two-sample equal variance, *p* < 0.05). Bars = 100 μm in (**A**) and 2 mm in (**D**).

We further checked the effect of auxin transport inhibitor 1-*N*-naphthylphthalamic acid (NPA) on *DR5:GUS* activity in WT and *cpi1-1* roots. Compared to the mock, obviously enhanced GUS staining was observed in both WT and *cpi1-1* root tips after NPA treatment (Figure 2A). Quantitative GUS activity assays showed that after NPA treatment, *DR5:GUS* activity was significantly increased in both WT and *cpi1-1* seedlings (Figure 2B), but *cpi1-1* mutant displayed lower fold induction compared with WT (Figure 2C), suggesting that it is less sensitive to NPA. This result is consistent with the previous report that *cpi1-1* displayed defective polar localization of the PIN2 auxin efflux protein [8]. Whereas higher concentrations of NPA (500 nM) inhibited root elongation of both WT and *cpi1-1* seedlings, there was no significant difference in relative root length between WT and *cpi1-1* (Figure 2D,E).

Together, these results suggest that other factors in addition to defective PAT contribute to the elevated auxin activity in *cpi1-1*.

### 2.2. The Expression of Auxin Biosynthesis Genes is Upregulated in cpi1-1

**Figure 3:**
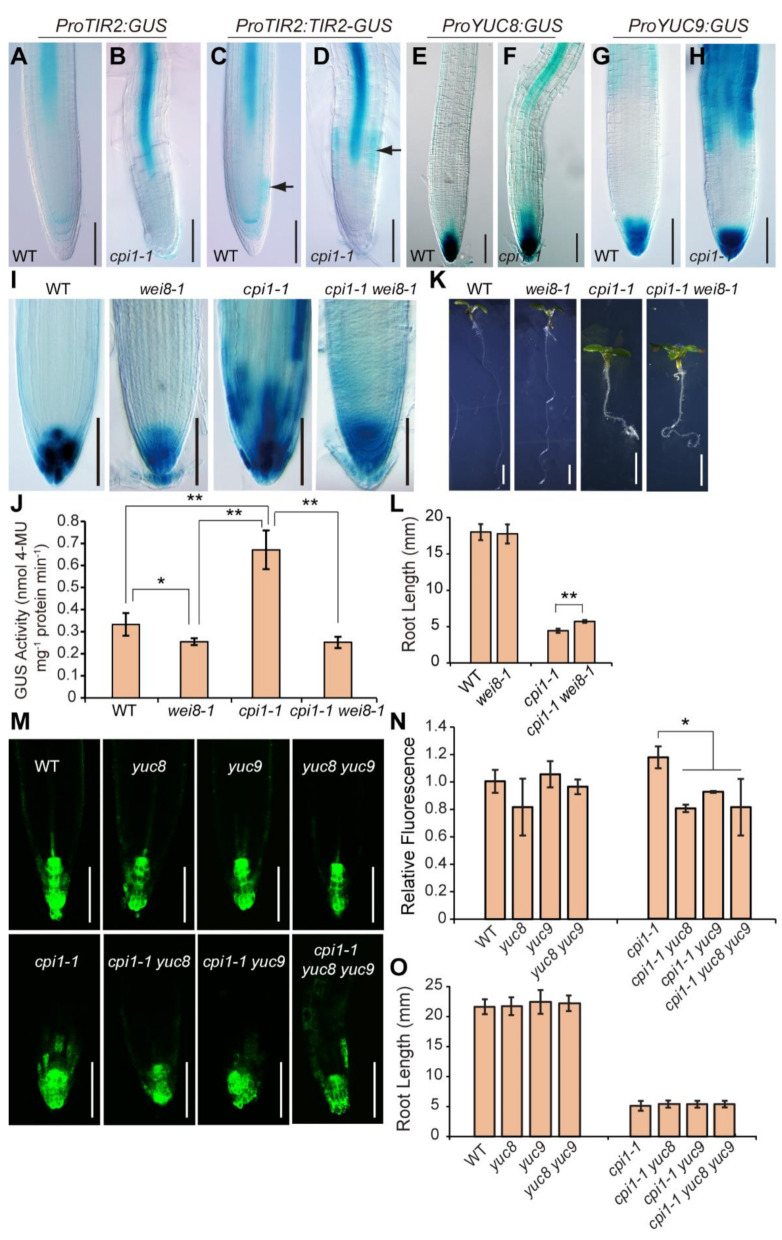
Auxin biosynthesis gene promoter activity is enhanced in *cpi1-1* and the *cpi1-1* short root phenotype is partially suppressed by the *wei8-1* mutation. (**A**–**H**) Expression patterns of *ProTIR2:GUS*, *ProTIR2:TIR2-GUS*, *ProYUC8:GUS*, and *ProYUC9:GUS* in 5-day-old WT and *cpi1-1* seedling roots. Shown are representative images of *n* = 3 independent experiments (employing 6 to 30 seedlings per experiment). The arrow in (**C**) indicates GUS staining in the root proximal meristem; The arrow in (**D**) indicates GUS staining in the elongation zone; (**I**) Expression patterns of *DR5:GUS* in root tips of 5-day-old WT, *wei8-1*, *cpi1-1*, and *cpi1-1 wei8-1* seedlings. Shown are representative images of three independent experiments (employing 18 to 32 seedlings per experiment); (**J**) Quantitative *DR5:GUS* activity assay in 5-day-old seedlings. The presented data are means ± SD of *n* = 3 independent experiments. * *p* < 0.05, ** *p* < 0.01 (one-way ANOVA with Tukey’s test); (**K**,**L**) Phenotypes (**K**) and root length (**L**) of 7-day-old seedlings. The presented data are means ± SD of *n* = 3 experiments (employing 13 to 28 seedlings per experiment). ** *p* < 0.01 (Student’s *t*-test, one-tailed, two-sample equal variance); (**M**,**N**) Expression patterns of *DR5_rev_:GFP* in root tips (**M**) and quantification of GFP fluorescence (**N**). The presented data are means ± SD of *n* = 3 independent experiments (employing 6 to 11 roots per experiment). * *p* < 0.05 (one-way ANOVA with Tukey’s test); (**O**) Root length of 7-day-old seedlings. The presented data are means ± SD of *n* = 3 independent experiments (employing 9 to 28 seedlings per experiment). No significant difference between *cpi1-1* single and *cpi1-1 yuc8* and *cpi1-1 yuc9* double and *cpi1-1 yuc8 yuc9* triple mutants by one-way ANOVA (*p* < 0.05). Bars = 100 μm in (**A**–**I**,**M**) and 2 mm in (**K**).

Since auxin biosynthesis and transport contribute to maintain appropriate auxin levels during plant development [37], we analyzed the expression patterns of auxin biosynthesis genes in *cpi1-1*. The *TAA1* (also known as *TRANSPORT INHIBITOR RESPONSE2*, *TIR2* or *WEAK ETHYLENE INSENSITIVE8*, *WEI8*) gene encodes tryptophan aminotransferase, which catalyzes the tryptophan (Trp) to indole-3-pyruvic acid (IPA) conversion [38,39,40]. In order to analyze *TAA1* expression patterns in *cpi1-1*, we crossed *ProTIR2:GUS* and *ProTIR2:TIR2-GUS* (a fusion of *TAA1*/*TIR2* cDNA sequence with the *GUS* reporter gene under control of the *TAA1*/*TIR2* promoter, which rescued the phenotype of the *tir2* mutant) [40] to *cpi1-1*. In 5-day-old WT seedlings, *ProTIR2:GUS* signal was detected in cotyledons, hypocotyl, and the root vascular tissue (Supplemental Figure S5A,B; Figure 3A). Compared to WT, obviously increased *ProTIR2:GUS* expression was observed in *cpi1-1* (Supplemental Figure S5C,D; Figure 3B). *ProTIR2:TIR2-GUS* expression was detected in cotyledons, hypocotyl, root vasculature, and root proximal meristem (Supplemental Figure S5E,F; Figure 3C). Consistent with the results of *ProTIR2:GUS*, *ProTIR2:TIR2-GUS* expression was also upregulated in *cpi1-1* (Supplemental Figure S5G,H; Figure 3D). In addition, the expression pattern of *ProTIR2:TIR2-GUS* was altered in the root tip of *cpi1-1*; it was not detected in the root proximal meristem, but was strongly expressed in the epidermal cells of the root elongation zone (Figure 3D). Since the *TAA1*/*TIR2* cDNA and genomic constructs displayed different expression patterns in the root tip [38,40,41], we also analyzed the expression pattern of *ProTAA1:GFP-TAA1* (a fusion of *TAA1* genomic sequence with the *GFP* reporter gene) [38] in *cpi1-1*. *ProTAA1:GFP-TAA1* was strongly expressed in the quiescent center (QC) cells and in columella initials, and its expression was not different between WT and *cpi1-1* (Supplemental Figure S6). Together, these results indicate that *CPI1* mutation leads to enhanced expression of *TAA1* in root vascular tissue and elongation zone, but do not affect its expression in the QC cells. These results are consistent with the previous report that *cpi1-1* roots have normal sized meristem but a shorter elongation zone [8].

YUC flavin monooxygenase proteins catalyze the conversion of indole-3-pyruvic acid to indole-3-acetic acid (IAA), which may be considered the rate-limiting step of the main auxin biosynthesis pathway [42,43,44]. To investigate whether the expression levels of *YUC* genes were altered in *cpi1-1*, we crossed *ProYUC2:GUS*, *ProYUC3:GUS*, *ProYUC4:GUS*, *ProYUC8:GUS*, and *ProYUC9:GUS* to *cpi1-1* [45,46]. In 5-day-old WT seedlings, *ProYUC2:GUS* was mainly expressed in cotyledons, the vascular tissue in the hypocotyl, in the shoot apical meristem, and in leaf primordia (Supplemental Figure S7A,B). Compared to WT, an enhanced expression of *ProYUC2:GUS* was observed in *cpi1-1* (Supplemental Figure S7C,D). *ProYUC3:GUS* was mainly expressed in the columella root cap cells, and its expression displayed no obvious difference between WT and *cpi1-1* (Supplemental Figure S7E,F)*. ProYUC4:GUS* expression was detected in the tips of the cotyledons, in leaf primordia, and in emerging young leaves, and its expression displayed no obvious difference between WT and *cpi1-1* (Supplemental Figure S7G–J). In 5-day-old WT seedlings, *ProYUC8:GUS* expression was detected mainly in the QC cells and in the columella root cap cells (Figure 3E; Supplemental Figure S8A–C). Of interest, the *ProYUC8:GUS* signals in the columella initials as well as in the L1 and L2 layers of the columella root cap cells was lower than that in the QC cells and in the L3 and L4 layers of the columella root cap cells (Supplemental Figure S8C). Compared to WT, increased staining of *ProYUC8:GUS* was observed in the root vascular tissues of *cpi1-1* (Figure 3F; Supplemental Figure S8E), but not in its root QC cells and columella root cap cells (Figure 3F; Supplemental Figure S8F). In 5-day-old WT seedlings, expression of *ProYUC9:GUS* was observed mainly in the columella root cap cells, in the root tip elongation zone, as well as in the root vasculature (Figure 3G; Supplemental Figure S8G–I). Compared to WT, *cpi1-1* displayed increased expression of *ProYUC9:GUS* in all the above-mentioned root tissues (Figure 3H; Supplemental Figure S8J–L).

We also analyzed the expression of auxin biosynthesis upstream genes including *ANTHRANILATE SYNTHASE α1* (*ASA1*, also known as *WEAK ETHYLENE INSENSITIVE2*, *WEI2*) and *ANTHRANILATE SYNTHASE β1* (*ASB1*, also known as *WEAK ETHYLENE INSENSITIVE7*, *WEI7*), which encode the α- and β-subunits of a key enzyme of tryptophan biosynthesis [47]. Compared to WT, enhanced expression of *ProASB1:GUS* was observed in *cpi1-1*, whereas expression of *ProASA1:GUS* was not obviously different (Supplemental Figure S9A).

To gain further evidence, RT-qPCR was performed on 7-day-old WT and *cpi1-1* seedlings. Consistent with the GUS staining results, *TAA1*, *YUC8*, and *YUC9* genes showed a significant upregulation in the *cpi1-1* mutant (Supplemental Figure S9B). *YUC2*, *ASA1*, and *ASB1* genes showed a slight, but not significant upregulation in the *cpi1-1* mutant (Supplemental Figure S9B).

Together, these results reveal that expression of a number of auxin biosynthesis pathway genes is upregulated in the *cpi1-1* mutant, and among them *TAA1* and *YUC8* genes show cell file specific upregulation.

### 2.3. Modulation of Auxin Biosynthesis Partially Rescues the Short Root Phenotype of cpi1-1

We then investigated whether mutation of auxin biosynthesis genes could reduce the *DR5:GUS* activity in *cpi1-1* and could rescue the short root phenotype of *cpi1-1*. We found that compared to the *cpi1-1* single mutant, *DR5:GUS* staining was obviously reduced in the *cpi1-1 wei8-1* double mutant (Figure 3I). GUS activity quantification results showed that the *cpi1-1 wei8-1* double mutant exhibited a similar GUS activity level as the *wei8-1* single mutant (Figure 3J). Consistently, mutation of *WEI8/TAA1* partially rescued the short root phenotype of *cpi1-1* (Figure 3K,L). We also compared the fluorescent signals of *DR5_rev_:GFP* in *cpi1-1* single, *cpi1-1 yuc8* and *cpi1-1 yuc9* double, and *cpi1-1 yuc8 yuc9* triple mutants. We found that *DR5_rev_:GFP* fluorescence was obviously reduced in *cpi1-1 yuc8* and *cpi1-1 yuc9* double and in *cpi1-1 yuc8 yuc9* triple mutants (Figure 3M,N). However, no significant difference of root length was observed in these mutants (Figure 3O), suggesting that additional *YUC* genes might play a role. *YUC2* and *YUC3* mutation also did not affect the hypocotyl length and root length of *cpi1-1* (Supplemental Figure S10).

**Figure 4:**
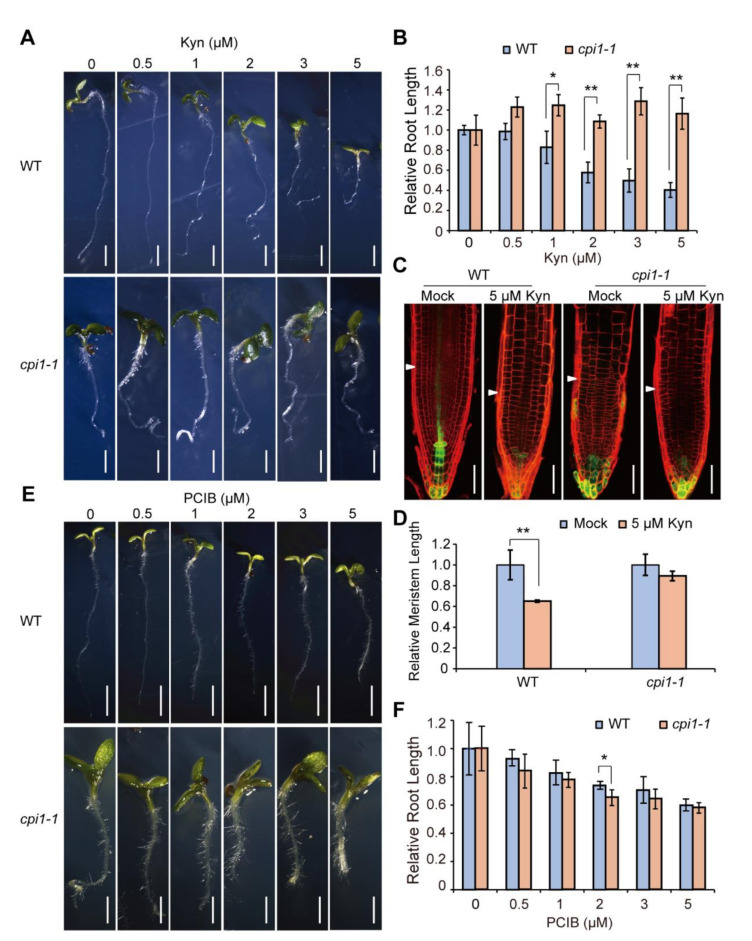
The *cpi1-1* short root phenotype is partially rescued by the L-Kynurenine (Kyn) auxin biosynthesis inhibitor, while *cpi1-1* responds normally to the *p*-Chlorophenoxyisobutyric acid (PCIB) auxin signaling inhibitor. (**A**,**E**) Phenotypes of WT and *cpi1-1* seedlings after treatment with various concentrations of Kyn (**A**) and PCIB (**E**) for 7 days; (**B**,**F**) Relative root length of 7-day-old WT and *cpi1-1* seedlings grown on MS medium supplemented with different concentrations of Kyn (**B**) and PCIB (**F**). MS medium supplemented with 0.1 mM HCl and 0.1% (*v*/*v*) ethanol, solvent for Kyn and PCIB, respectively, served as blank controls. The presented data are means ± SD of *n* = 3 independent experiments (employing 8 to 55 seedlings per experiment. * p < 0.05, ** p < 0.01 (one-way ANOVA with Tukey’s test); (**C**) Meristem architectures of 7-day-old WT and *cpi1-1* seedling roots grown on MS medium supplemented with or without 5 μM Kyn; (**D**) Relative meristem zone length of 7-day-old seedling roots. The presented data are means ± SD of *n* = 3 independent experiments (employing 18 to 28 roots per experiment). ** *p* < 0.01 (Student’s *t*-test, one-tailed, two-sample equal variance). Bars = 2 mm in (**A**,**E**, top panel), 1 mm in (**A**,**E**, bottom panel), and 50 μm in (**C**).

L-Kynurenine (Kyn) is an auxin biosynthesis inhibitor that inhibits TAA1 activity [48]. We thus checked the effect of Kyn on *cpi1-1* root growth by growing WT and *cpi1-1* seedlings in Murashige and Skoog (MS) media supplemented with a range of concentrations of Kyn (0, 0.5, 1, 2, 3, and 5 μM) for 7 days. WT root growth was inhibited by these Kyn treatments especially at higher Kyn concentrations (Figure 4A,B). For example, after treatment with 2, 3, and 5 μM Kyn, the WT root length was approximately 58%, 50%, and 40% of the mock, respectively. In contrast, *cpi1-1* root growth was partially rescued by Kyn treatment, particularly at 0.5, 1, and 3 μM Kyn, the *cpi1-1* root length was increased by more than 21% (Figure 4A,B). Previous research showed that Kyn treatment inhibited root growth by blocking root meristem function [41]. Therefore, we measured the root meristem size of WT and *cpi1-1* seedlings after Kyn treatment. The results showed that size of the meristem zone of WT root was significantly reduced under 5 uM Kyn treatment, but no statistical difference was observed in the root meristem zone length of *cpi1-1* mutant (Figure 4C,D). These results suggest that *cpi1-1* is less sensitive to Kyn treatment.

Together, these results demonstrate that genetic and pharmacological inhibition of auxin biosynthesis can partially rescue the short root phenotype of *cpi1-1*.

### 2.4. cpi1-1 Responds Normally to the Auxin Signaling Inhibitor

*p*-Chlorophenoxyisobutyric acid (PCIB) is an auxin signaling inhibitor [49]. To investigate whether *CPI1* mutation has an effect on auxin signaling, we grew WT and *cpi1-1* seedlings in MS media supplemented with different concentrations of PCIB (0, 0.5, 1, 2, 3, and 5 μM) for 7 days. We found that root growth of both WT and *cpi1-1* were inhibited by PCIB treatment, and their relative root length showed no significant difference (Figure 4E,F), indicating that *cpi1-1* roots respond normally to the auxin signaling inhibitor. These data suggest that *cpi1-1* mutation may not affect the auxin signaling.

### 2.5. Cycloeucalenol Application Increases the Expression of Auxin Biosynthesis Genes

**Figure 5:**
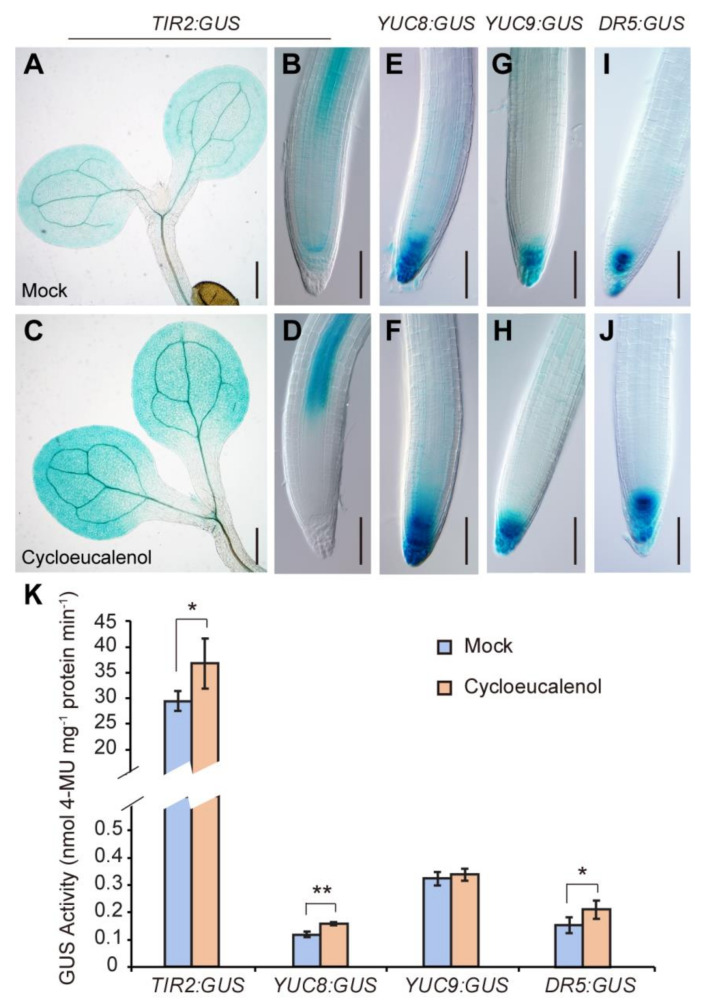
Cycloeucalenol enhances auxin biosynthesis genes and *DR5:GUS* auxin response reporter expression. (**A**–**D**) Expression patterns of *ProTIR2:GUS* in shoots (**A**,**C**) and root tips (**B**,**D**) of 5-day-old WT seedlings grown on MS medium containing 0.1% acetone solvent (mock) or 1 μM cycloeucalenol. Shown are representative images of three independent experiments (employing 11 to 26 seedlings per experiment); (**E**–**J**) Expression patterns of *ProYUC8:GUS* (**E**,**F**), *ProYUC9:GUS* (**G**,**H**), and *DR5:GUS* (**I**,**J**) in root tips of 5-day-old WT seedlings grown on MS medium containing 0.1% (*v*/*v*) acetone solvent (mock) or 1 μM cycloeucalenol. Shown are representative images of three independent experiments (employing 10 to 26 seedlings per experiment); (**K**) GUS activity of *ProTIR2:GUS*, *ProYUC8:GUS*, *ProYUC9:GUS*, and *DR5:GUS* 5-day-old seedlings. The presented data are means ± SD of *n* = 3 independent experiments. * *p* < 0.05, ** *p* < 0.01 (Student’s *t*-test, one-tailed, two-sample equal variance). Bars = 400 μm in (**A**,**C**) and 100 μm in (**B**,**D**–**J**).

Previously, Men et al. (2008) demonstrated that sterol composition was markedly altered in the *cpi1-1* plants. Cycloeucalenol, a minor sterol component in WT (1.0 μg/g fresh weight (fw)), was detected as a major sterol compound in *cpi1-1* (50.3 μg/g fw) [8]. By contrast, sitosterol, the predominant sterol in WT (122.3 μg/g fw), was reduced to trace amounts in *cpi1-1* (2.5 μg/g fw). 24-methylcholesterol (campesterol) and stigmasterol, the other two major sterols in WT (22.4 and 10.3 μg/g fw, respectively), were undetectable in *cpi1-1* [8]. We wondered whether these alterations of sterol composition caused the upregulation of auxin biosynthesis in *cpi1-1*. To address this, we performed cycloeucalenol treatment on *ProTIR2:GUS*, *ProYUC8:GUS*, *ProYUC9:GUS*, and *DR5:GUS* expressing seedlings. Upon cycloeucalenol treatment (1 μM), the GUS staining of *ProTIR2:GUS* and *DR5:GUS* was obviously stronger than that of the mock (0.1% acetone (*v/v*), the solvent for cycloeucalenol) (Figure 5A–D,I,J). The GUS signals were also slightly enhanced in the *ProYUC8:GUS* and *ProYUC9:GUS* lines treated with cycloeucalenol (Figure 5E–H). To gain further evidence, we performed quantitative GUS activity assay. The results showed that after cycloeucalenol treatment, the GUS activity in *ProTIR2:GUS*, *ProYUC8:GUS*, and *DR5:GUS* seedlings was significantly increased, but no statistical difference was observed in *ProYUC9:GUS* with or without cycloeucalenol treatment (Figure 5K). In RT-qPCR analysis, *YUC8* and *TAA1* genes also showed significant upregulation after cycloeucalenol treatment, whereas *YUC9* gene showed a slight, but not significant upregulation upon cycloeucalenol treatment (Supplemental Figure S11A). RT-qPCR was also performed to determine whether cycloeucalenol affects the expression of PAT genes. The results showed that the expression of *PIN* genes and *AUX1/LAX* genes was not significantly different between mock and cycloeucalenol treatments (Supplemental Figure S11B). These results indicate that while cycloeucalenol can contribute to upregulation of the expression of auxin biosynthesis genes, it does not affect *PIN* and *AUX1/LAX* gene expression.

### 2.6. Sitosterol Application Decreases DR5:GUS and ProYUC8:GUS Expression and Partially Rescues the cpi1-1 Short Root Phenotype

**Figure 6:**
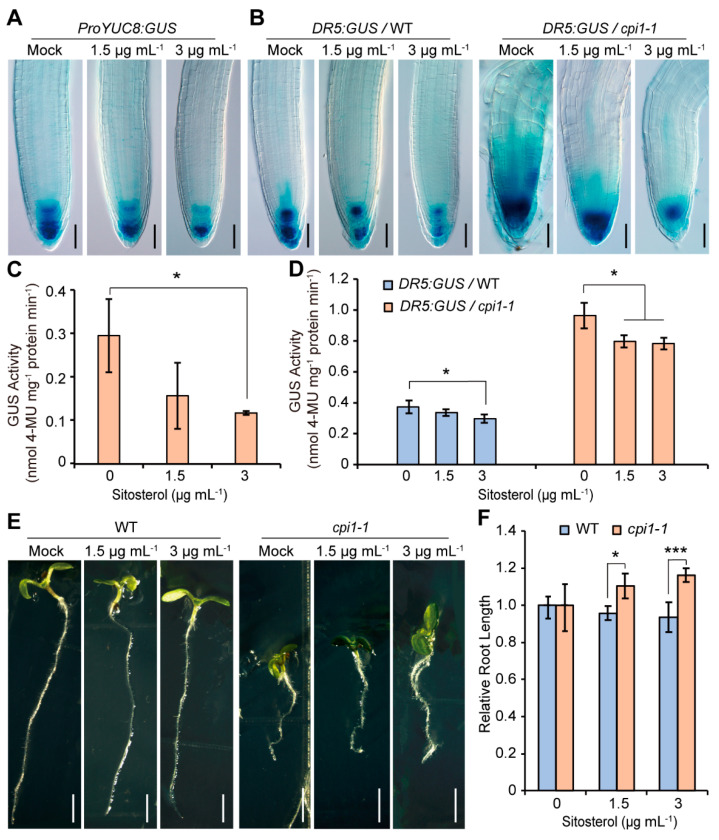
Sitosterol represses the expression of *ProYUC8:GUS* and *DR5:GUS*, and partially rescues the short root phenotype of *cpi1-1*. (**A**) Expression of *ProYUC8:GUS* treated with or without sitosterol. The images are representative of *n* = 3 independent experiments employing 9 to 30 seedlings per experiment; (**B**) Expression of *DR5:GUS* in WT and *cpi1-1* seedlings treated with or without sitosterol. The images are representative of *n* = 3 independent experiments employing 14 to 30 seedlings per experiment; (**C**,**D**) Quantitative GUS activity assay of *ProYUC8:GUS* (**C**) and *DR5:GUS* (**D**) in 5-day-old seedlings. Data are shown as means ± SD of *n* = 3 independent experiments. * *p* < 0.05 (one-way ANOVA with Tukey’s test); (**E**,**F**) Phenotypes (**E**) and relative root length (**F**) of WT and *cpi1-1* seedlings grown on MS medium supplemented with different concentrations of sitosterol. Data are shown as means ± SD of *n* = 3 experiments (employing 12 to 45 roots per experiment). * *p* < 0.05, *** *p* < 0.001 (Student’s *t*-test, one-tailed, two-sample equal variance). Bars = 100 μm in (**A**,**B**) and 1 mm in (**E**).

To investigate whether sitosterol affects auxin biosynthesis gene expression, RT-qPCR was performed on 5-day-old WT seedlings grown on MS medium supplemented with or without sitosterol. The results showed that the expression of *YUC8* was reduced by sitosterol treatment, whereas the expression of *YUC9* and *TAA1* was not significantly different between mock and sitosterol treatments (Supplemental Figure S12A). To gain further evidence, we compared the expression of *ProTIR2:GUS*, *ProYUC8:GUS*, and *ProYUC9:GUS* between mock and sitosterol treatments. Consistent with the RT-qPCR results, after sitosterol treatment the GUS staining and GUS activity of *ProYUC8:GUS* was obviously reduced (Figure 6A,C), whereas *ProTIR2:GUS* and *ProYUC9:GUS* displayed no obvious difference between mock and sitosterol treatments (Supplemental Figure S12B). Consistently, sitosterol repressed expression of *DR5:GUS* in both WT and *cpi1-1* roots (Figure 6B,D). We then investigated whether sitosterol was involved in regulating auxin transport by examining the transcript levels of auxin transporters after sitosterol treatment. The results revealed that, except for *PIN7*, the expression of none of the other *PIN* and *AUX/LAX* genes examined was altered after sitosterol treatment (Supplemental Figure S12C). We then investigated whether the root length of *cpi1-1* was influenced by sitosterol. Application of sitosterol slightly repressed the root growth of WT, but partially rescued the short root phenotype of *cpi1-1* (Figure 6E,F).

To determine whether stigmasterol and cholesterol affect the root growth of *cpi1-1,* we measured the root length of 7-day-old WT and *cpi1-1* seedlings grown on MS medium supplemented with or without stigmasterol or cholesterol. Stigmasterol repressed the root elongation of both WT and *cpi1-1*, while cholesterol showed no detectable influence on root growth (Supplemental Figure S13).

**Figure 7:**
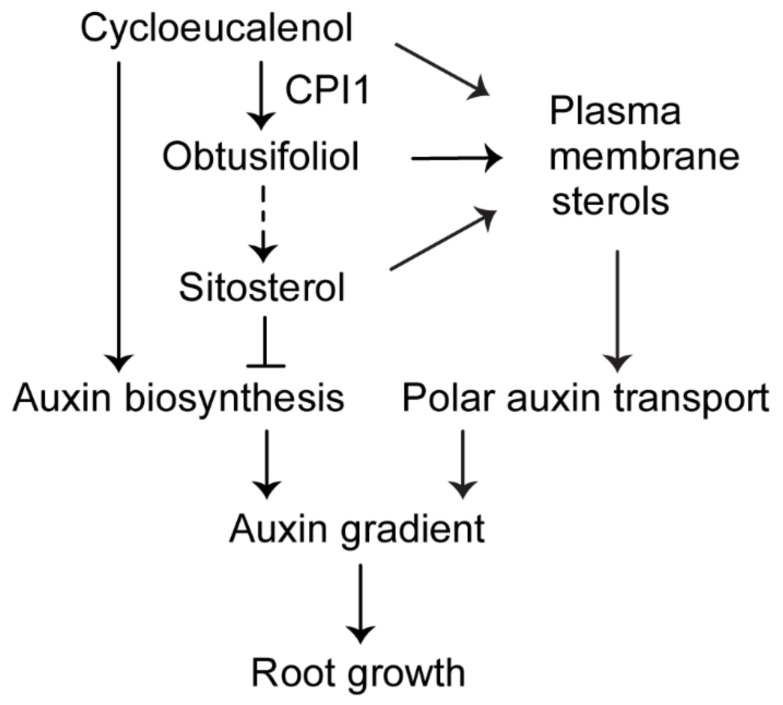
A working model for the role of CPI1 in Arabidopsis root growth. In the plant sterol biosynthesis pathway, CPI1 catalyzes the conversion of cycloeucalenol into obtusifoliol, which is further metabolized to sitosterol. Cycloeucalenol upregulates the expression of auxin biosynthesis genes; by contrast, sitosterol represses the expression of these genes. Normal content of cycloeucalenol and sitosterol is important for optimal auxin biosynthesis, and correct plasma membrane sterol composition is required for normal polar auxin transport. Auxin biosynthesis and polar auxin transport cooperatively establish an optimal auxin gradient in the root tip to regulate root growth.

Taken together, our results suggest that cycloeucalenol and sitosterol have opposite effects on auxin biosynthesis and activity (Figure 7).

## 3. Discussion

### 3.1. Auxin Activity and Auxin Biosynthesis are Increased in the cpi1-1 Mutant

The *cpi1-1* mutant displayed enhanced and ectopic *DR5:GUS* expression in the root tip [8], suggesting that either auxin signaling is enhanced in the *cpi1-1* mutant or its root tip accumulates more auxin. We found that the *cpi1-1* mutant responds normally to the auxin signaling inhibitor, so it is unlikely that auxin signaling is enhanced in *cpi1-1*. The *DR5:GUS* expression patterns in *cpi1-1* root tip resembled that of *pin2-T* mutant and polar PIN2 localization was disrupted in *cpi1-1* root tip epidermis and cortex cells [8], suggesting that defective PAT caused auxin accumulation in the *cpi1-1* root tip. However, our genetic experiments showed that *DR5:GUS* signals in *cpi1-1 pin2-T* double mutant root tips was stronger than either *cpi1-1* or *pin2-T* single mutant, indicating an additive effect. This finding suggests that defective PAT alone cannot explain the increased levels of auxin in the roots of *cpi1-1* mutants. Indeed, we found that expression of auxin biosynthesis genes including *TAA1*, *YUC8*, and *YUC9* was upregulated in the *cpi1-1* mutant. These results suggest that altered sterol composition in the *cpi1-1* mutant not only affects PAT but also affects auxin biosynthesis.

A recent report showed that in rice, low membrane sterol levels compromised the interaction between the ethylene receptor OsERS2 and the inhibitory kinase OsCTR2 in the ER membrane, thus activates the ethylene response [50]. It has been known that ethylene upregulates auxin biosynthesis [51,52,53,54]. Thus, reduced membrane sterol content might upregulate auxin biosynthesis through activation of ethylene signaling. The *cpi1-1* mutant displays an almost complete replacement of the usual Δ^5^-sterols including sitosterol, campesterol, and stigmasterol by 9β,19-cyclopropyl sterols, but its total sterol content is not reduced compared to that of the WT (285 μg/g fw in *cpi1-1* versus 190 μg/g fw in WT) [8]. The next step should analyze whether ethylene signaling is affected in the *cpi1-1* mutant to determine if altered sterol composition also has an effect on ethylene signaling.

### 3.2. Cycloeucalenol Might Play a Role in Plant Development

In vertebrates and fungi, lanosterol synthase (LAS) cyclizes 2,3-oxidosqualene to the tetracyclic lanosterol, which is further metabolized to tetracyclic cholesterol and ergosterol, respectively, whereas in plants, 2,3-oxidosqualene is mainly cyclized to the pentacyclic cycloartenol (which contains a 9β,19-cyclopropane ring) by cycloartenol synthase (CAS) [3,55,56]. After C-24 methylation and C-4 demethylation, cycloartenol is converted to cycloeucalenol [3]. Then, CPI1 catalyzes the opening of the 9β,19-cyclopropane ring of cycloeucalenol, and converts it to tetracyclic obtusifoliol [29], which is further metabolized to tetracyclic end-products such as sitosterol and stigmasterol (Supplemental Figure S1). Why plants use this two-enzyme route instead of cyclizing directly to tetracyclic sterols and whether those 9β,19-cyclopropyl sterols have physiological roles are long-standing questions that remain to be answered. Several 9β,19-cyclopropyl sterols, including cycloartenol, 24-methylene cycloartanol, and cycloeucalenol, are indeed present as minor sterol compounds in plants [2,6,7,8,9,10,57]. During male gametophyte development, the tetrads have similar sterol species as vegetative organs, but mature pollen cells accumulate esterified 9β,19-cyclopropyl sterols [58,59]. CAS1 loss of function leads to male-specific transmission defect [55]. These results suggest that 9β,19-cyclopropyl sterols might have a role in male gametophyte development and transmission. Interestingly, cycloeucalenol was found to be the major newly synthesized sterol during pollen cells germination and its accumulation increased with pollen tube elongation [58,59]. Furthermore, inhibition of cycloeucalenol synthesis inhibited pollen tube elongation [58]. Mutants upstream of *CPI1* such as *hmg1* also showed defects in pollen cells germination and pollen tube elongation [60]. These findings strongly suggest that cycloeucalenol is required for pollen cells germination and pollen tube growth. How cycloeucalenol contributes to pollen cells germination and pollen tube elongation is so far unknown. Like pollen tubes, root hairs also grow in a polarized fashion. Whether cycloeucalenol is also involved in root hair growth is unknown. Recently, it has been reported that induced accumulation of a 9β,19-cyclopropyl sterol, 4-carboxy-4methyl-24-methylenecycloartanol (CMMC), by loss-of-function of *ERGOSTEROL BIOSYNTHETIC PROTEIN28* (*ERG28*) led to phenotypes resemble of those caused by defective PAT, including inhibited root growth, pin-like inflorescence, and fused leaves [10]. Furthermore, exogenous application of CMMC inhibited PAT [10]. Since CMMC was not detectable in WT at normal growth conditions, further investigations are required to determine at what circumstances it is released to regulate PAT. In this study, we found that increased content of cycloeucalenol in *cpi1-1* was associated with enhanced auxin biosynthesis and response. Furthermore, in vitro application of cycloeucalenol upregulated the expression of auxin responsive reporter *DR5:GUS* and auxin biosynthesis genes such as *TAA1/TIR2* and *YUC8*. These results imply that cycloeucalenol might play a role in plant development and growth by regulating auxin biosynthesis. Further investigations are required to understand how cycloeucalenol upregulates auxin biosynthesis.

### 3.3. Sitosterol Modulates Auxin Biosynthesis and Response

Previous studies have shown that a number of sterol mutants exhibit aberrant auxin response and polar auxin transport due to altered sterol composition [8,9,19,21,22,25,27]. However, whether a certain sterol component has a function in regulating auxin activity is largely unknown. Sitosterol, campesterol, and stigmasterol are the most abundant end-products of the plant sterol biosynthesis pathway [2,3,4,5]. It has been found that in vitro application of sitosterol and stigmasterol enhanced the expression of genes involved in cell expansion and cell division [61]. Exogenous application of sitosterol and stigmasterol but not campesterol partially rescued the abnormal auxin-induced lateral root development in the *smt2 smt3* double mutant [26]. Further research demonstrated that application of sitosterol restored the *DR5:GUS* signal gradient in the lateral root tips of the *smt2 smt3* mutant, suggesting that directional auxin transport was amended by sitosterol [26]. These results were consistent with the altered sterol profiles in the *smt2 smt3* mutant, as in this mutant the sitosterol and stigmasterol content were reduced to trace amount, whereas the campesterol content was increased [9]. Similarly, in *cpi1-1*, sitosterol content was reduced to trace amount, whereas stigmasterol and campesterol were undetectable [8]. We found that the aberrant sterol profile in *cpi1-1* was associated with increased auxin biosynthesis in addition to defective PAT. Further analysis showed that sitosterol application reduced the expression of auxin biosynthesis genes and an auxin responsive reporter and partially rescued the short root phenotype of *cpi1-1*. These results suggest that sitosterol functions in regulating auxin activity by inhibiting auxin biosynthesis. How sitosterol regulates the expression of auxin biosynthesis genes remains to be determined. In animals, cholesterol stimulates the autoprocessing of the precursor protein of the Hedgehog (Hh) morphogen important for embryo development. Cholesterol covalently attaches to the N-terminus of the mature Hh protein, which is essential for the long-range activity of Hh [62,63]. Recently, Xiao et al. (2017) found that the Hh signaling pathway downstream protein Smoothened (SMO) was also covalently modified by cholesterol, which activates SMO [64]. In plants, no sitosterol-binding protein has been identified so far. Interestingly, a recent study found that the early gravistimulation induced ROSY1 protein contains a MD2-lipid binding domain and specifically binds stigmasterol in vitro [65]. Mutation of *ROSY1* leads to decreased basipetal auxin transport and enhanced gravitropic response [65]. Screens for sitosterol-binding proteins should provide insights for understanding the roles of sitosterol in plant growth and development.

Overall, our findings suggest that cycloeucalenol upregulates, whereas sitosterol downregulates auxin biosynthesis. The altered sterol composition in *cpi1-1* leads to enhanced auxin biosynthesis and defective polar auxin transport, resulting in an enhanced, ectopic auxin gradient in the *cpi1-1* root tip accompanied by altered root growth. Hence, our findings provide an initial mechanistic basis for future studies on the molecular mechanisms by which sterols influence auxin biosynthesis in plants.

## 4. Materials and Methods

### 4.1. Plant Materials and Growth Conditions

*Arabidopsis thaliana* (Arabidopsis) ecotype Columbia (Col-0) and Landsberg *erecta* (L*er*) were used as the wild type (WT). The following mutants were used in this study: *cpi1-1* transposon insertion mutant in the L*er* background [8] and *cpi1-1* out-crossed to the Col-0 background [66], T-DNA insertion mutants *pin2-T* [8], *aux1-T* [34], *yuc2* (SALK_030199) [45], *wei8-1* [38], and *yuc8* (SALK_096110) and *yuc9* (SAIL_871_G01) [67,68] are in Col-0 background. These mutants were verified by PCR-based genotyping using primers listed in Supplemental Table S1. The following transgenic reporter lines were used in this study: *DR5:GUS* [32,33], *DR5_rev_:GFP* [69], *ProPIN1:GUS* and *ProPIN7:GUS* [70], *ProPIN3:GUS* [71], *ProPIN4:GUS* [72], *PIN7-GFP* [73], *ProASA:GUS* and *ProASB:GUS* [47], *ProTAA1:GFP-TAA1* [38], *ProTIR2:GUS* and *ProTIR2:TIR2-GUS* [40], *ProYUC2:GUS* and *ProYUC4:GUS* [45], and *ProYUC8:GUS* and *ProYUC9:GUS* [46]. Arabidopsis seeds were surfaced sterilized first with 70% (*v*/*v*) ethanol (Sangon Biotech, Shanghai, China) for 5 min, then with 1% (*v*/*v*) NaClO (Sangon Biotech, Shanghai, China) for 10 min, washed five times with sterile water, incubated for 3–4 days at 4 °C, and sowed on Murashige Skoog (MS) medium (pH 5.8) (Duchefa Biochemie, Haarlem, Netherlands) supplemented with 1% (*w*/*v*) sucrose (Sangon Biotech, Shanghai, China) and solidified by 0.8% (*w*/*v*) agar (Duchefa Biochemie, Haarlem, Netherlands). The seeds were germinated and grown at 22 °C under a 16 h light/8 h dark photoperiod with 90–110 μmol m^−2^ s^−1^ illumination.

### 4.2. GUS Staining

*GUS* expression of various genotypes were detected by incubating 5-day-old seedlings of various plant lines in a staining solution (0.5 mg mL^−1^ 5-bromo-4-chloro-3-indolyl-β-d-glucuronic acid, 50 mM sodium phosphate, 0.5 mM ferricyanide, and 0.1% (*v*/*v*) Triton X-100, pH 7.0) at 37 °C for various hours (indicated below). After incubation, the samples were first fixed in ethanol:acetic acid (2:1) for 6 h, then were cleared in a clearing solution of 8:3:1 (*w*/*v*/*v*) chloral hydrate:distilled water:glycerol. The samples were observed using an Olympus BX63 (Olympus Corporation, Tokyo, Japan) microscope. The incubation hours for different transgenic lines in the GUS staining solution are as follows: 12 h (WT and mutants in Figure 1, Figure 2 and Figure 3) or 3 h (the sterols treatment experiments in Figure 5 and Figure 6) for *DR5:GUS*, 3 h for *ProTIR2:GUS* and *ProTIR2:TIR2-GUS*, 36 h for *ProPIN1:GUS*, 6 h for *ProPIN3:GUS*, 1 h for *ProPIN4:GUS*, *ProPIN7:GUS*, *ProASA1:GUS*, and *ProYUC8:GUS*, and 2 h for *ProASB1:GUS* and *ProYUC9:GUS*. Analysis was done in biological triplicate. 5-bromo-4-chloro-3-indolyl-β-d-glucuronic acid was purchased from Bio Basic Inc (Toronto, Canada), other reagents used in this part were purchased from Sangon Biotech (Shanghai, China).

### 4.3. Fluorescence Microscopy

GFP fluorescence was observed either using a fluorescent microscope (Olympus BX63, Olympus Corporation, Tokyo, Japan) or by a confocal laser scanning microscope (Leica TCS SP5, Wetzlar, Germany) using 488 nm excitation filter with 505–550 nm band pass. Images were processed with Adobe Photoshop CS5 and assembled in Adobe Illustrator CS4.

### 4.4. Auxin Inhibitor Treatments

For 1-*N*-naphthylphthalamic acid (NPA) treatments, WT and *cpi1-1/+* seeds were germinated and grown for 7 days on MS medium supplemented with different concentrations of NPA (0, 250, and 500 nM) for 7 days. NPA was dissolved in DMSO (Sangon Biotech, Shanghai, China). For L-Kynurenine (Kyn) treatments, WT and *cpi1-1/+* seeds were germinated on MS medium supplemented with different concentrations of Kyn (0, 0.5, 1, 2, 3, and 5 μM) for 7 days. Kyn was dissolved in 0.1 M HCl (Sangon Biotech, Shanghai, China). For *p*-chlorophenoxyisobutyric acid (PCIB) treatments, WT and *cpi1-1/+* seeds were germinated on MS medium supplemented with different concentrations of PCIB (0, 0.5, 1, 2, 3, and 5 μM) for 7 days. PCIB was dissolved in absolute ethanol. NPA, Kyn, and PCIB were purchased from Sigma-Aldrich (Shanghai, China).

### 4.5. RNA Extraction and Reverse Transcription Quantitative PCR (RT-qPCR)

Total RNA was extracted from 7-day-old seedlings by TRIzol according to the manufacturer’s instructions (TransGen Biotech, Beijing, China). For RT-qPCR analysis, approximately 2.5 μg of total RNA was reverse-transcribed into cDNA using the EasyScript First-Strand cDNA Synthesis SuperMix (TransGen Biotech, Beijing, China). One microliter of each cDNA sample was mixed with 7.5 µl SYBR Green Real-Time PCR Master Mix (DBI Bioscience, Shanghai, China), and then analyzed on a fluorescent quantitative PCR machine (Eppendorf, Hamburg, Germany). The *TAP42 INTERACTING PROTEIN OF 41 KDA* (*TIP41*, AT4G34270) gene was used as an internal control. The relative transcription level was calculated by the 2^−∆∆Ct^ (Ct, cycle threshold) value. Primers used in the RT-qPCR analysis are listed in Supplemental Table S1.

### 4.6. Sterol Treatment

For cycloeucalenol and sitosterol treatments, *ProTIR2:GUS*, *ProYUC8:GUS*, *ProYUC9:GUS*, and *DR5:GUS* seeds were germinated and grown for 5 days on cycloeucalenol- or sitosterol-containing MS medium (pH 5.8) solidified by 0.8% (*w*/*v*) agar. Seedlings were then collected for GUS staining. The concentrations of sterols used were according to previous publications [26,60]. Cycloeucalenol (purchased from BioBioPha, Kunming, China) was dissolved in absolute acetone (Sangon Biotech, Shanghai, China) and added to the MS medium at 1 μM final concentration. The same MS medium supplemented with 0.1% (*v*/*v*) acetone was used as control. Sitosterol (purchased from TCI Shanghai, Shanghai, China) was dissolved in absolute chloroform (Sangon Biotech, Shanghai, China) and added to the MS medium at 1.5 or 3 μg mL^−^^1^ final concentrations. The same MS medium supplemented with 0.1% (*v*/*v*) chloroform was used as control. For stigmasterol and cholesterol treatments, WT and *cpi1-1* seeds were germinated and grown for 7 days on stigmasterol- or cholesterol-containing MS medium (pH 5.8) solidified by 0.8% (*w*/*v*) agar. Stigmasterol (purchased from TCI Shanghai, Shanghai, China) and cholesterol (purchased from Sigma-Aldrich, Shanghai, China) were dissolved in absolute chloroform and added to the MS medium at 1 or 10 μM final concentrations, respectively. The same MS medium supplemented with 0.1% (*v*/*v*) chloroform was used as control.

### 4.7. Statistical Analyses

Three independent repetitions were conducted for all experiments. Statistical analyses were performed by using one-tailed Student’s *t*-test or using one-way analysis of variance (ANOVA) with Tukey’s test. All values are presented as means ± SD. Significant differences are noted as follows: * *p* < 0.05, ** *p* < 0.01, and *** *p* < 0.001.

### 4.8. Accession Numbers

Sequence data from this article for the cDNA and genomic DNA of *CPI1* can be found in The Arabidopsis Information Resource database (https://www.arabidopsis.org/) under accession number At5g50375. T-DNA insertion lines used for mutant analyses were as follows: *pin2-T* (SALK_091142), *aux1-T* (SALK_020355), *yuc2* (SALK_030199), *wei8-1* (CS16407), *yuc8* (SALK_096110), and *yuc9* (SAIL_871_G01).

## Data Availability

The data presented in this study are available in article and supplementary material.

## Supplementary Materials

Supplementary Materials can be found at https://www.mdpi.com/1422-0067/22/1/437/s1: Figure S1. Plant sterol biosynthetic pathway; Figure S2. *DR5:GUS* expression in shoot, root tips and lateral root primordia of 2-week-old WT and *cpi1-1* seedlings; Figure S3. *ProPIN1:GUS* and *ProPIN3:GUS* expression in seedling shoots and roots; Figure S4. *ProPIN4:GUS*, *ProPIN7:GUS* and *PIN7-GFP* expression in WT and *cpi1-1* roots and relative transcript levels of *PIN* genes; Figure S5. *ProTIR2:GUS* and *ProTIR2:TIR2-GUS* expression patterns in WT and *cpi1-1* shoots; Figure S6. *ProTAA1:GFP-TAA1* expression in 5-day-old WT and *cpi1-1* seedling roots; Figure S7. *ProYUC2:GUS*, *ProYUC3:GUS* and *ProYUC4:GUS* expression patterns in 5-day-old seedlings; Figure S8. *ProYUC8:GUS* and *ProYUC9:GUS* expression patterns in 5-day-old WT and *cpi1-1* seedling shoot, root vasculature, and root tip; Figure S9. *ProASA1:GUS* and *ProASB1:GUS* expression patterns in WT and *cpi1-1* seedlings and relative transcript levels of auxin biosynthesis genes; Figure S10. Mutation of *YUC2* or *YUC3* does not rescue the short root and short hypocotyl phenotypes of *cpi1-1*; Figure S11. Relative transcript levels of auxin biosynthesis genes and polar auxin transport genes upon cycloeucalenol treatment; Figure S12. Relative transcript levels of auxin biosynthesis and polar auxin transport genes upon sitosterol treatment and *ProTIR2:GUS* and *ProYUC9:GUS* expression in seedling roots; Figure S13. Effects of stigmasterol and cholesterol on WT and *cpi1-1* root growth. Table S1. List of primers used in this study.

## Abbreviations

BRs	brassinosteroids
fw	fresh weight
IAA	indole-3-acetic acid
Kyn	L-Kynurenine
NPA	1-*N*-naphthylphthalamic acid
PAT	polar auxin transport
PCIB	*p*-chlorophenoxyisobutyric acid
QC	quiescent center
SBIs	sterol biosynthetic intermediates
WT	wild type
