# Supplementary material for: Membrane Sterol Composition in Arabidopsis thaliana Affects Root Elongation via Auxin Biosynthesis
Source: Int J Mol Sci. 2021 Jan 4;22(1):437. doi: 10.3390/ijms22010437 (PMC7794993; doi:10.3390/ijms22010437)
Supplement: Supplementary file 1 [file ijms-22-00437-s001.pdf]

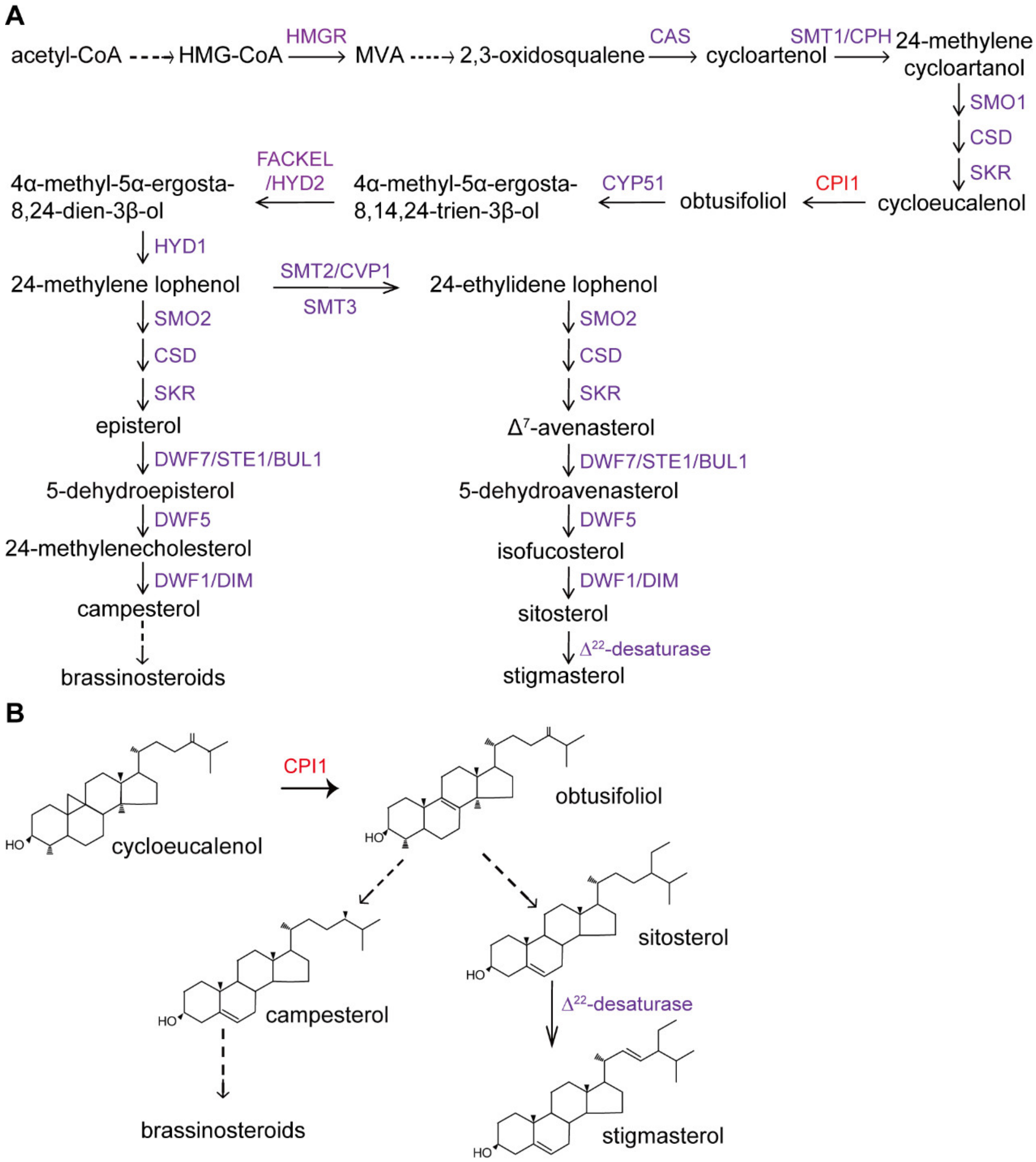

Fig. S1. Plant sterol biosynthetic pathway. **(A)** Plant sterol biosynthetic pathway; **(B)** CPI1-related intermediates and end-products. Arrows with dashed lines represent multiple steps. HMG-CoA, 3-hydroxy-3-methylglutaryl coenzyme A; HMGR, 3-hydroxy-3-methylglutaryl coenzyme A reductase; MVA, mevalonic acid; CAS, cycloartenol synthase; SMT1/CPH, C-24 methyl transferase; SMO1, sterol 4*α*-methyl oxidase1; CSD, 4*α*-carboxysterol-C3-dehydrogenase/C4-decarboxylase; SKR, sterone 3-keto reductase; CPI1, cyclopropylsterol isomerase1; CYP51, sterol C-14-demethylase; FACKEL/HYD2, sterol C-14 reductase; HYD1, Δ^8-Δ^7-sterol isomerase; SMT2/CVP1, C-28 methyl transferase; SMT3, C-28 methyl transferase; SMO2, sterol 4*α*-methyl oxidase2; DWF7/STE1/BUL1, Δ^7-sterol C-5-desaturase; DWF5, Δ^7-sterol C-7 reductase; DWF1/DIM, C-24 reductase.

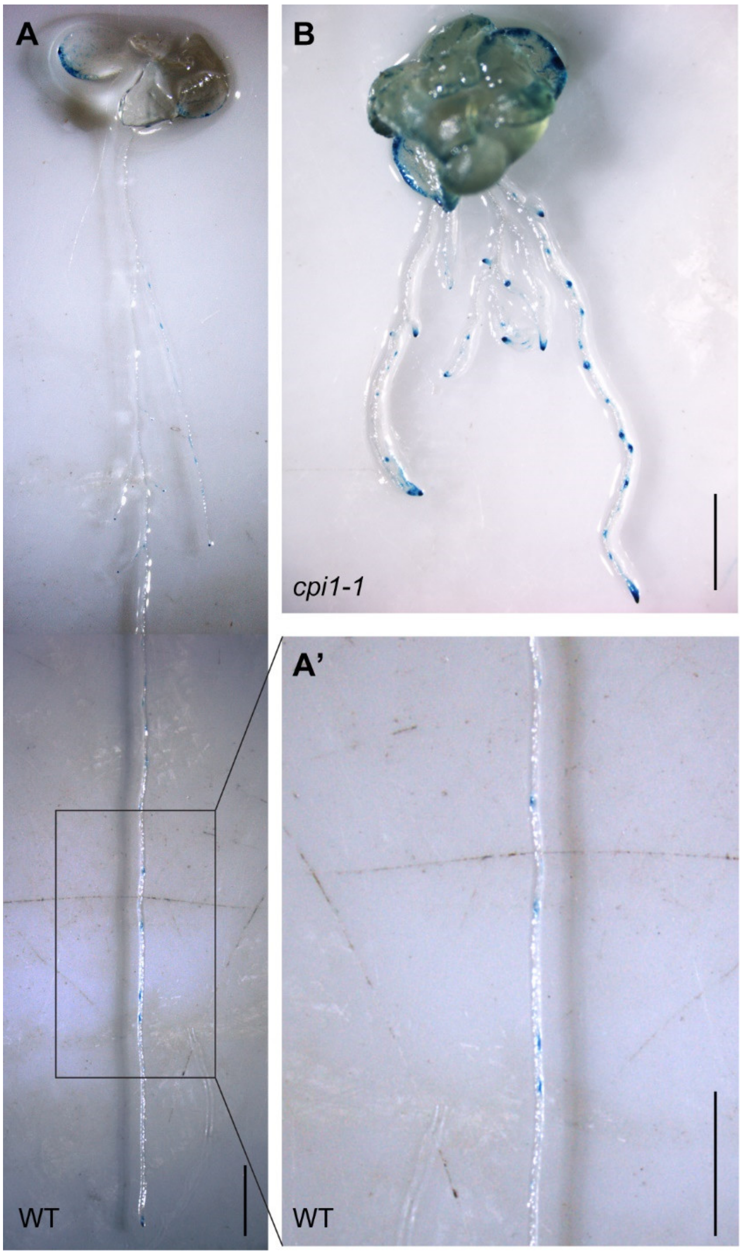

Fig. S2. *DR5:GUS* expression in shoot, root tips and lateral root primordia of 2-week-old WT (A and A') and *cpi1-1* (B) seedlings. A' are higher magnification image of the root region in A. Shown are representative images of n = 3 independent experiments, employing 6 to 10 seedlings per experiment. Bars = 2 mm (A and B) and 8 mm (A').

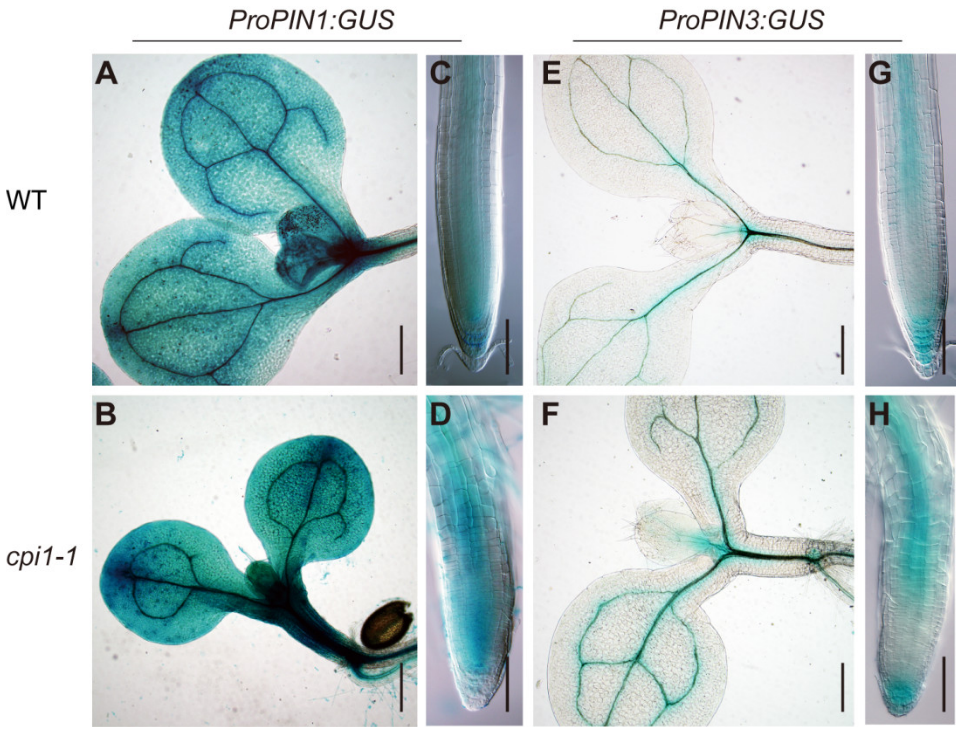

Fig. S3. *ProPIN1:GUS* and *ProPIN3:GUS* expression in seedling shoots and roots. **(A-H)** GUS staining of *ProPIN1:GUS* (A-D) and *ProPIN3:GUS* (E-H) in 5-day-old wild type (WT) and *cpi1-1* seedlings. Shown are representative images of n = 3 independent experiments, employing 7 to 31 seedlings per experiment. Bars = 400 μm (A, B, E and F) and 100 μm (C, D, G and H).

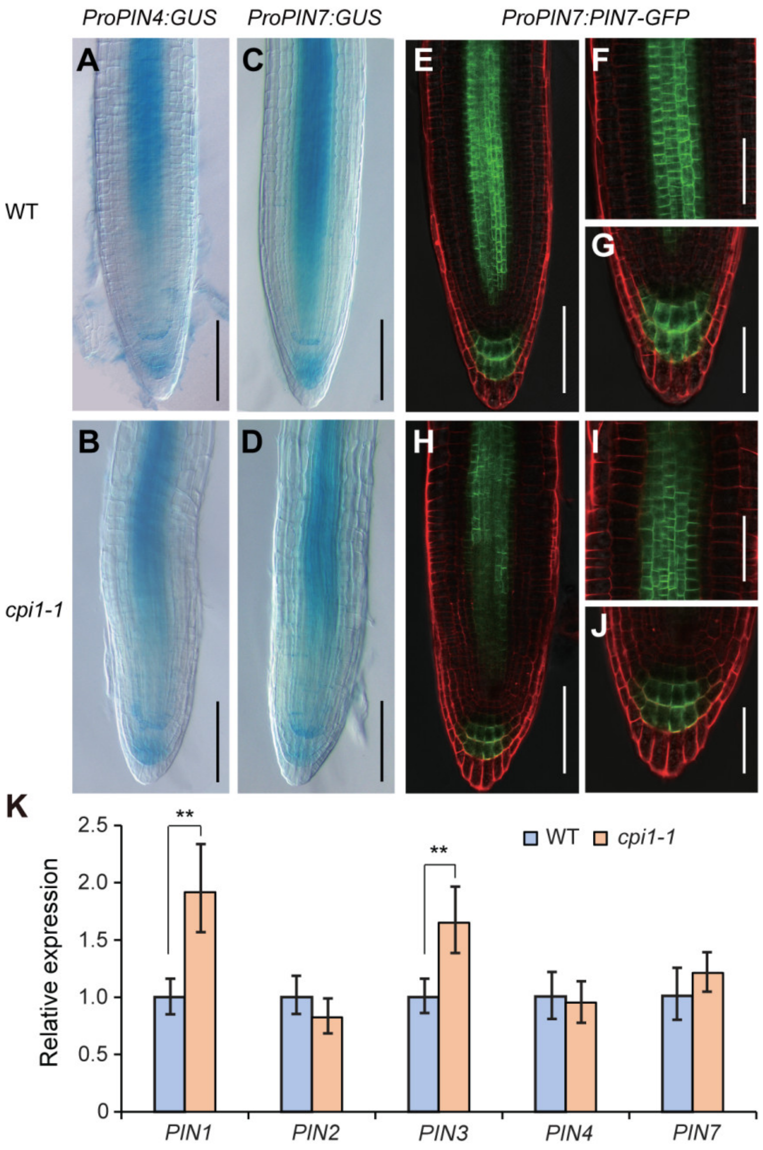

Fig. S4. *ProPIN4:GUS*, *ProPIN7:GUS* and *PIN7-GFP* expression in WT and *cpi1-1* roots and relative transcript levels of *PIN* genes. **(A-D)** GUS staining of *ProPIN4:GUS* (A and B) and *ProPIN7:GUS* (C and D) in 5-day-old WT (A and C) and *cpi1-1* (B and D) seedling roots; **(E-J)** GFP signals of *PIN7-GFP* in 5-day-old WT (E-G) and *cpi1-1* (H-J) seedling roots. F and G are higher magnification images of the stele and columella regions, respectively in E; I and J are higher magnification images of the stele and columella regions, respectively in H. Shown are representative images of n = 3 independent experiments, employing 9 to 26 roots per experiment. Bars = 100 μm (A- E and H) and 50 μm (F, G, I and J); **(K)** Relative transcript levels of polar auxin transport genes. The *TAP42 INTERACTING PROTEIN OF 41 KDA* (*TIP41*, AT4G34270) gene was used as an internal control. The presented data are means ± SD of n = 3 independent experiments. $^{**}P < 0.01$ (Student's *t*-test, one-tailed, two-sample equal variance).

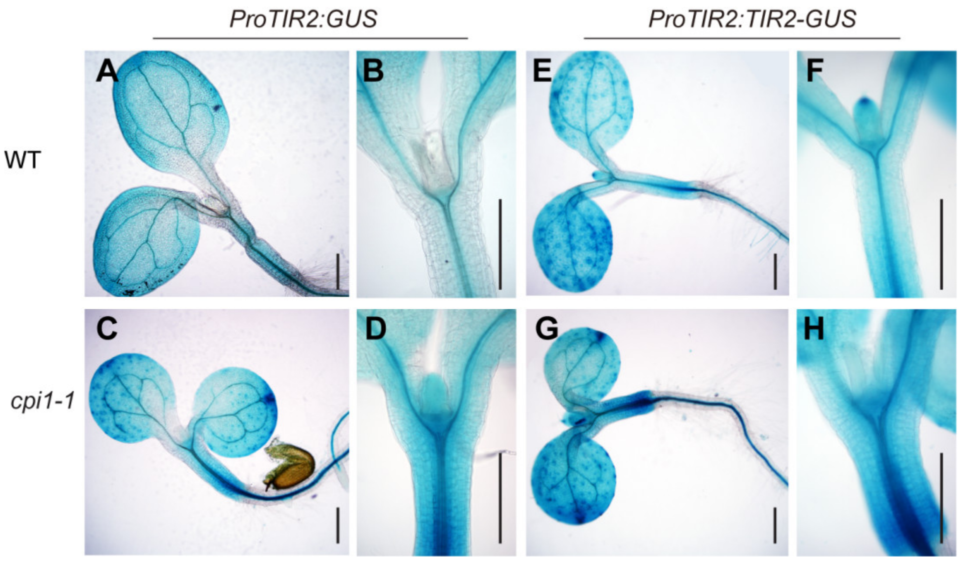

Fig. S5. *ProTIR2:GUS* and *ProTIR2:TIR2-GUS* expression patterns in WT and *cpi1-1* shoots. **(A-D)** Expression patterns of *ProTIR2:GUS* in 5-day-old WT (A and B) and *cpi1-1* (C and D) seedling shoots; **(E-H)** Expression patterns of *ProTIR2:TIR2-GUS* in 5-day-old WT (E and F) and *cpi1-1* (G and H) seedling shoots. Shown are representative images of n = 3 independent experiments, employing 6 to 30 seedlings per experiment. Bars = 400 μm.

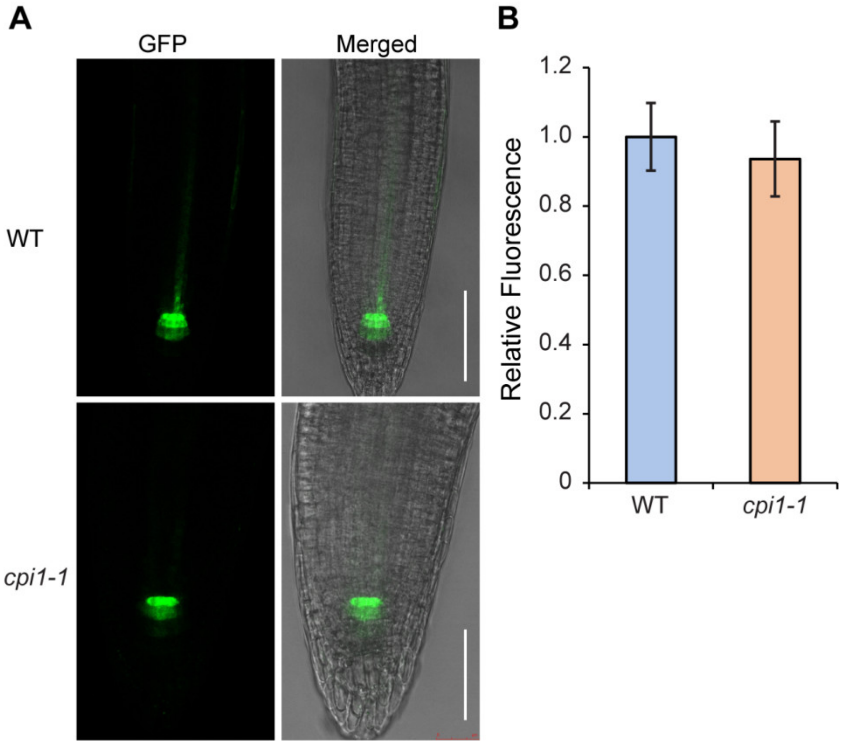

Fig. S6. *ProTAA1:GFP-TAA1* expression in 5-day-old WT and *cpi1-1* seedling roots. **(A and B)** Expression patterns of *ProTAA1:GFP-TAA1* in root tips (A) and quantification of GFP fluorescence (B). The presented data are means ± SD of n = 3 independent experiments (employing 4 to 22 roots per experiment). No significant difference by Student's *t*-test (one-tailed, two-sample equal variance, $P < 0.05$). Bars = 100 μm.

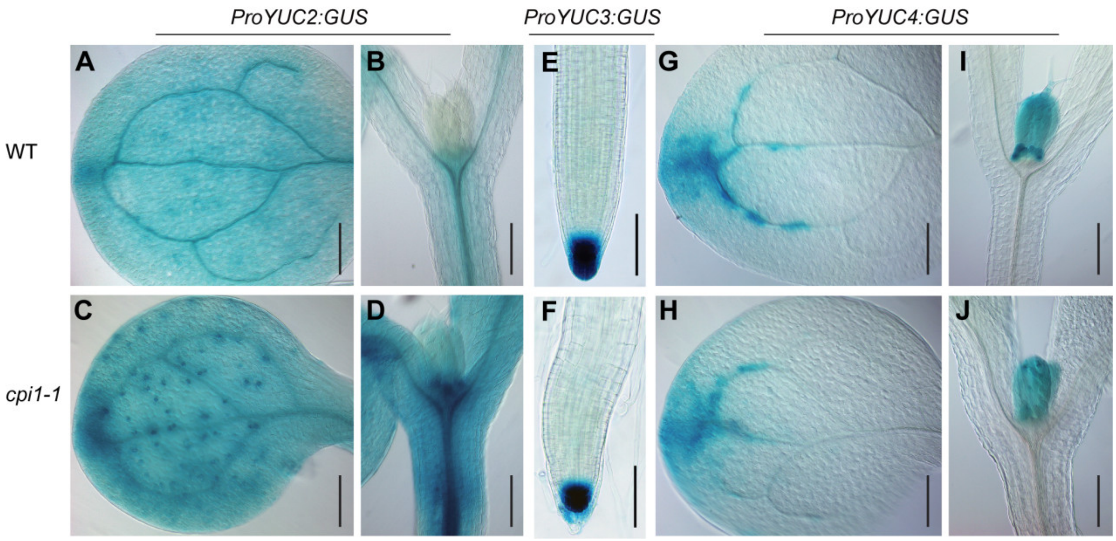

Fig. S7. *ProYUC2:GUS*, *ProYUC3:GUS* and *ProYUC4:GUS* expression patterns in 5-day-old seedlings. **(A-D)** GUS staining of *ProYUC2:GUS* in 5-day-old WT (A and B) and *cpi1-1* (C and D) seedling cotyledon (A and C), shoot meristem and apical part of the hypocotyl (B and D); **(E-F)** GUS staining of *ProYUC3:GUS* in 5-day-old WT (E) and *cpi1-1* (F) seedling roots; **(G-J)** GUS staining of *ProYUC4:GUS* in 5-day-old WT (G and I) and *cpi1-1* (H and J) seedling cotyledon (G and H), shoot meristem and apical part of the hypocotyl (I and J). Shown are representative images of n = 3 independent experiments, employing 8 to 14 seedlings per experiment. Bars = 200 μm in (A-D and G-J) and Bars = 100 μm in (E and F).

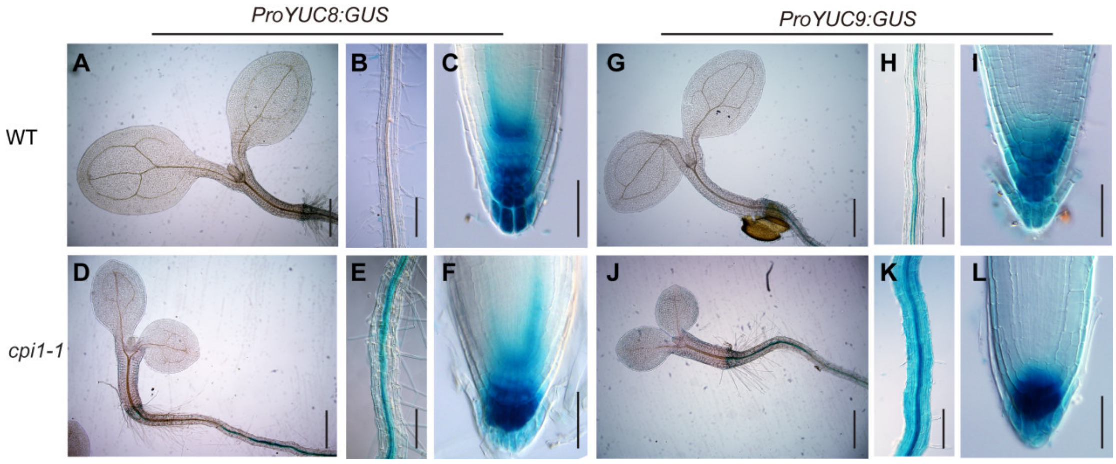

Fig. S8. *ProYUC8:GUS* and *ProYUC9:GUS* expression patterns in 5-day-old WT and *cpi1-1* seedling shoot, root vasculature, and root tip. **(A-F)** Expression patterns of *ProYUC8:GUS* in 5-day-old WT (A-C) and *cpi1-1* (D-F) seedlings; **(G-L)** Expression patterns of *ProYUC9:GUS* in 5-day-old WT (G-I) and *cpi1-1* (J-L) seedlings. Shown are representative images of n = 3 independent experiments, employing 6 to 22 seedlings per experiment. Bars = 0.5 mm in (A, D, G, and J), 200 μm in (B, E, H, and K), and 50 μm in (C, F, I, and L).

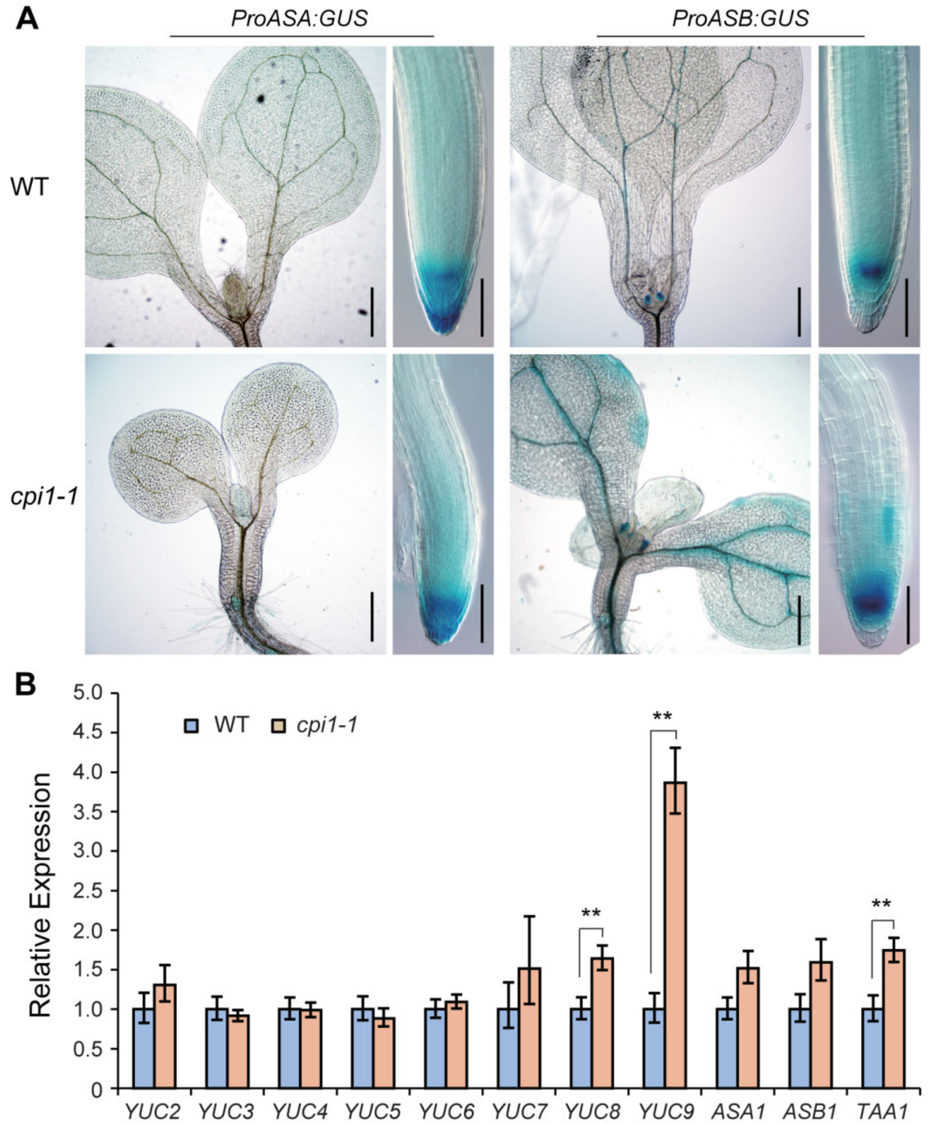

Fig. S9. *ProASA1:GUS* and *ProASB1:GUS* expression patterns in WT and *cpi1-1* seedlings and relative transcript levels of auxin biosynthesis genes. **(A)** Expression patterns of *ProASA1:GUS* and *ProASB1:GUS* in shoots and roots of 5-day-old WT and *cpi1-1* seedlings (n = 3 independent experiments, employing 8 to 24 seedlings per experiment). Bars = 400 μm in the shoot images and 100 μm in the root images; **(B)** Relative transcript levels of auxin biosynthesis genes. The *TIP41* gene was used as an internal control. The presented data are means ± SD of n = 3 independent experiments. $^{**}P < 0.01$ (Student's *t*-test, one-tailed, two-sample equal variance).

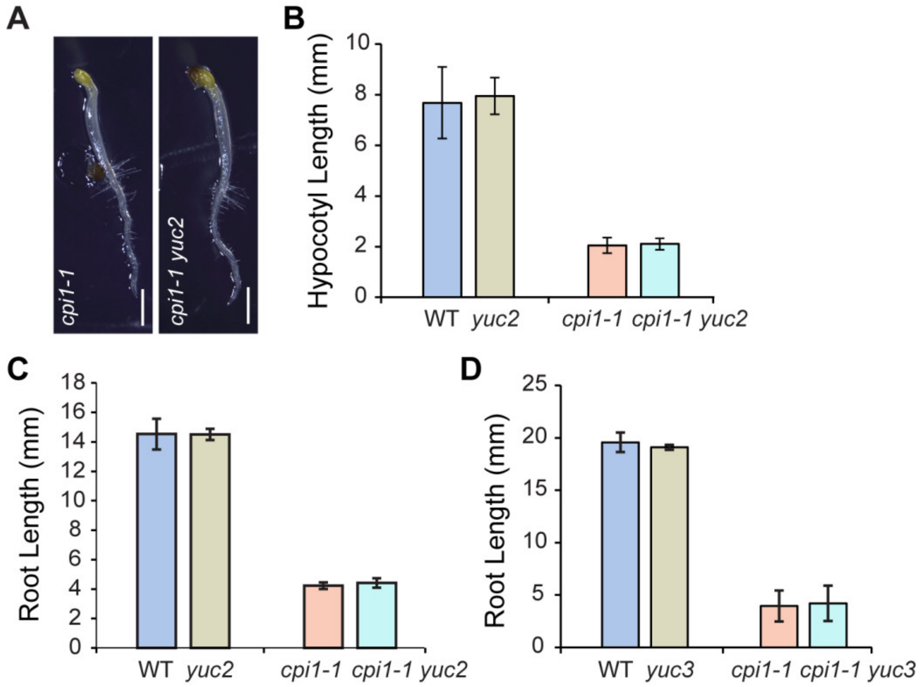

Fig. S10. Mutation of *YUC2* or *YUC3* does not rescue the short root and short hypocotyl phenotypes of *cpi1-1*. The presented data are means ± SD of n = 3 independent experiments (employing 9 to 47 seedlings per experiment). No significant difference between *cpi1-1* single and *cpi1-1 yuc2* and *cpi1-1 yuc3* double mutants by Student's *t*-test (one-tailed, two-sample equal variance, $P < 0.05$).

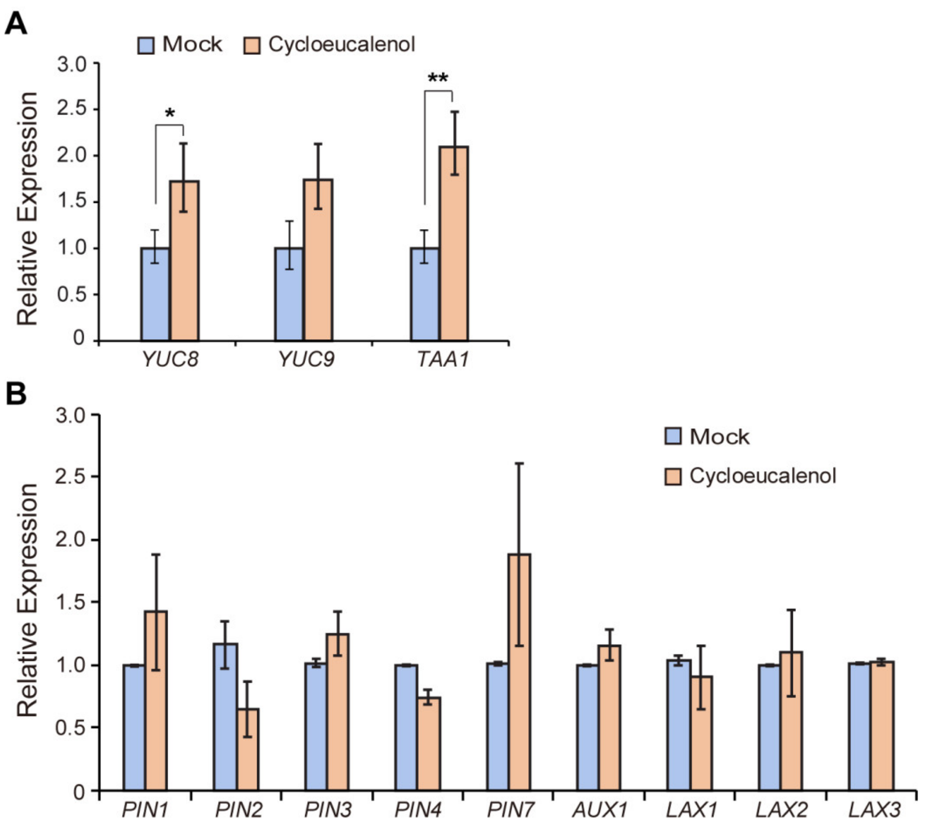

Fig. S11. Relative transcript levels of auxin biosynthesis genes **(A)** and polar auxin transport genes **(B)** upon cycloeucalenol treatment. WT seeds were germinated on MS medium supplemented with 0.1% (v/v) acetone (mock) or 1 μM cycloeucalenol for 7 days. Then these 7-day-old seedlings were collected for RT-qPCR analysis. The *TIP41* gene was used as an internal control. The presented data are means ± SD of n = 3 independent experiments. **P* < 0.05; ***P* < 0.01 (Student's *t*-test, one-tailed, two-sample equal variance).

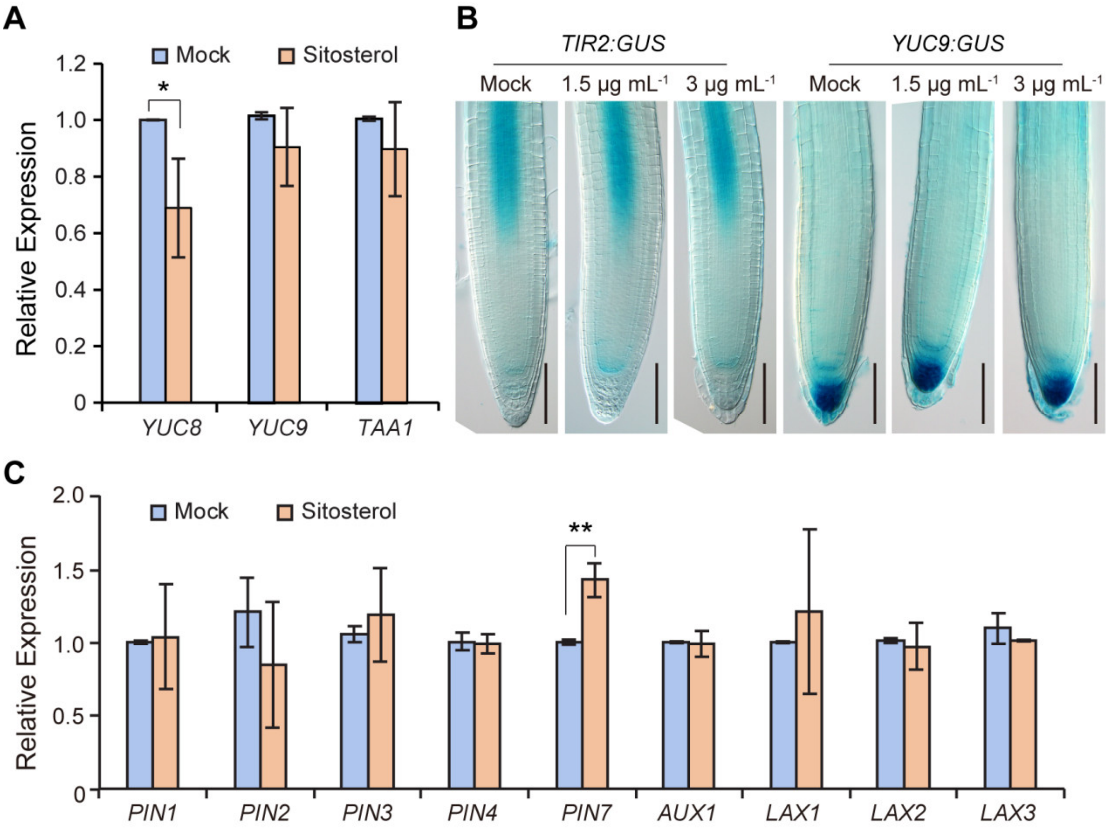

Fig. S12. Relative transcript levels of auxin biosynthesis and polar auxin transport genes upon sitosterol treatment and *ProTIR2:GUS* and *ProYUC9:GUS* expression in seedling roots. **(A and C)** Relative transcript levels of auxin biosynthesis genes (A) and polar auxin transport genes (C) upon sitosterol treatment. WT seeds were germinated on MS medium supplemented with 0.1% (v/v) chloroform (mock) or 3 μg mL^{-1} of **s**itosterol for 7 days. Then these 7-day-old seedlings were collected for RT-qPCR analysis. The *TIP41* gene was used as an internal control. The presented data are means ± SD of n = 3 independent experiments. *$P < 0.05$, **$P < 0.01$ (Student's *t*-test, one-tailed, two-sample equal variance); **(B)** Expression patterns of *ProTIR2:GUS* and *ProYUC9:GUS* after treatment with various concentrations of sitosterol for 5 days. The images are representative of n = 3 independent experiments employing 7 to 17 roots per experiment.

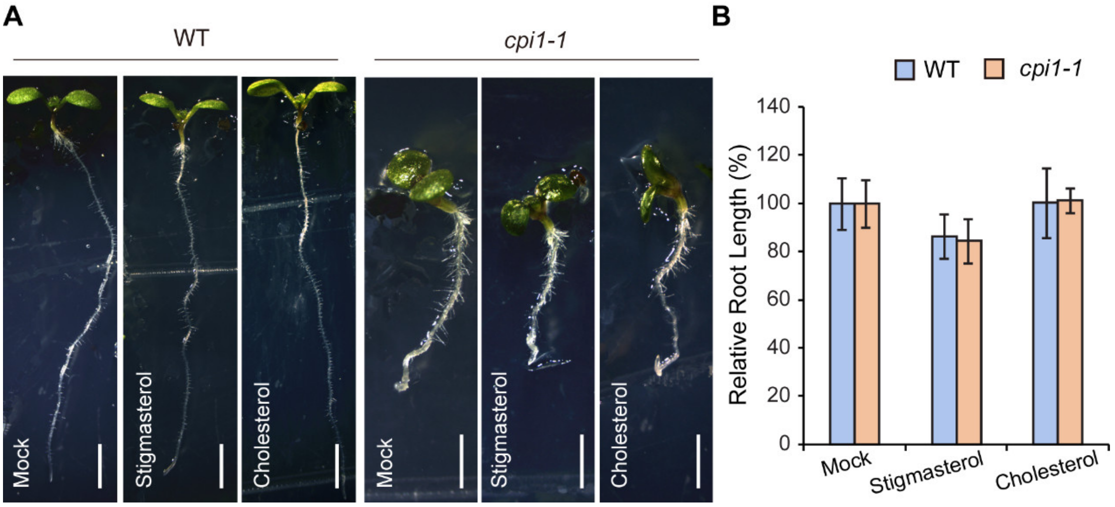

Fig. S13. Effects of stigmasterol and cholesterol on WT and *cpi1-1* root growth. **(A and B)** Phenotypes (A) and relative root length (B) of 7-day-old seedlings grown on MS medium supplemented with 1 μM stigmasterol or 10 μM cholesterol. The presented data in (B) are means ± SD of n = 3 independent experiments (employing 15 to 69 roots per experiment). No significant difference between mock and treatment in either WT or *cpi1-1* mutant by Student's *t*-test (one-tailed, two-sample equal variance, $P < 0.05$). Bars = 2 mm.

Table S1. List of primers used in this study.

Purpose	Primer name	Sequence (5′ to 3′)
Genotyping	LBa1	TGGTTCACGTAGTGGGCCATC
	SAIL-LB1	TTTTCAGAAATGGATAAATAGCC
	Ds5-1	ACGGTCGGGAAACTAGCTCTAC
	*cpi1-1*_LP	CTCGGCTCACTCACTCACACT
	*cpi1-1*_RP	CTGCCGAGATAATGCTGTGCTT
	*aux1-T*_LP	GGTTTACTAGGAAGCTGGACTGC
	*aux1-T*_RP	TGGACCTGAATGTTTCACACC
	*pin2-T*_LP	GGTCAACGAGTGGAGCAAGT
	*pin2-T*_RP	GCCATTCCAAGACCAGCATCA
	*wei8-1*_LP	CATCAGAGAGACGGTGGTGAAC
	*wei8-1*_RP	GCTTTTAATGAGCTTCATGTTGG
	*yuc2*_1031F	GCTCAAGTGGTTTCCAGTGCA
	*yuc2*_1828R	GCATCCACTACTACCTTTCTAC
	*yuc8*_-176F	ACGCCACATGGGATCTCTTC
	*yuc8*_401R	GACTCACTCTTCGACACGGTC
	*yuc9*_LP	CTTTACTCGACCGGGCTAGG
	*yuc9*_RP	TTTACCGAGGGAGATTATGGG
RT-qPCR	TIP41_qF	GTATGAAGATGAACTGGCTGACAAT
	TIP41_qR	ATCAACTCTCAGCCAAAATCGCAAG
	PIN1_qF	TTGCTGAGCTCCTACTTAAG
	PIN1_qR	GGCATGGCTATGTTCAGTCT
	PIN2_qF	AAGTCACGTACATGCATGTG
	PIN2_qR	AGATGCCAACGATAATGAGTG
	PIN3_qF	GAGTTACCCGAACCTAATCA
	PIN3_qR	TTACTGCGTGTCGCTATAGT
	PIN4_qF	ACCACTTAACTAGAAACTTCA
	PIN4_qR	TCATTGCTTGTGGGAACTCT
	PIN7_qF	TCTAGTTGCGTTCCACTAATC
	PIN7_qR	CGGTAAAACATATGCCACCA
	AUX1_qF	GCCTCCGCTCGTCAGAAT
	AUX1_qR	ACGGTGGTGTAAAGCGGAGA

	LAX1_qF	TACTCCGAGACCTTCCAACTACG
	LAX1_qR	TCCACCGCCACCACTTCC
	LAX2_qF	GGAGAACGGTGAGAAAGC
	LAX2_qR	TCAGATAGCTTAGATTTGATGTC
	LAX3_qF	GGTTTATTGGGCGTTTGG
	LAX3_qR	TGATTGGTCCGAAAAAGG
	YUC2_qF	ACTCGCCACGGGTTACAAAA
	YUC2_qR	CAATGGCTGCACCAAGCAAT
	YUC3_qF	GACATCGGAGCGTTACCCAA
	YUC3_qR	GCCTCTCCTTTCCATCCGTT
	YUC4_qF	ACCGACCTTTTAGGCCTTCG
	YUC4_qR	TCACGGCTTGCGTCACTTTA
	YUC5_qF	TTCAACGAGTGTGTCCAGTCTGCT
	YUC5_qR	TCTCTGGAACAACTTTCTCCGCGT
	YUC6_qF	TATACGCGGTCGGATTCACA
	YUC6_qR	CCACCACAATCACTCTCACT
	YUC7_qF	TACCTTGAGTCCTACGCTACCC
	YUC7_qR	ACCACCAAAATCTTCTAAACCCT
	YUC8_qF	CGTCTCAAGCTTCACCTTCC
	YUC8_qR	AGCCACTGGTCTCATCGAAC
	YUC9_qF	GACGGAGTTTGACGGAGAAG
	YUC9_qR	CCCTCGGTAAAACATGAACC
	ASA1_qF	GTAGAGAAGCTTATGAACATCGA
	ASA1_qR	GGTGCACCACTAACTGTTCCCAC
	ASB1_qF	GGGGAAGAGTCGTAGAGATGTCT
	ASB1-qR	CTGGCAGAGATTGTATGTGAAGC
	TAA1_qF	GATGAAGAATCGGTGGGAGA
	TAA1_qR	CGGACATGCTTCTTGTCAGA
